# Convergent evolution of [*D*-Leucine^1^] microcystin-LR in taxonomically disparate cyanobacteria

**DOI:** 10.1186/1471-2148-13-86

**Published:** 2013-04-19

**Authors:** Tânia Keiko Shishido, Ulla Kaasalainen, David P Fewer, Leo Rouhiainen, Jouni Jokela, Matti Wahlsten, Marli Fátima Fiore, João Sarkis Yunes, Jouko Rikkinen, Kaarina Sivonen

**Affiliations:** 1 Department of Food and Environmental Sciences, Division of Microbiology and Biotechnology, University of Helsinki, Viikki Biocenter (Viikinkaari 9), P.O. Box 56, Helsinki, FIN-00014, Finland; 2 Department of Biosciences, University of Helsinki, Viikki Biocenter (Viikinkaari 1), P.O. Box 65, Helsinki, FIN-00014, Finland; 3 Center for Nuclear Energy in Agriculture, University of São Paulo, Avenida Centenário 303, Piracicaba, SP, 13400-970, Brazil; 4 Unidade de Pesquisas em Cianobactérias, Federal University of Rio Grande, Av. Itália km 8 - Caixa Postal 474, Rio Grande, RS, 96.201-900, Brazil

**Keywords:** Adenylation domain, Phylogeny, Substrate specificity, Gene conversion, Point mutations, Recombination

## Abstract

**Background:**

Many important toxins and antibiotics are produced by non-ribosomal biosynthetic pathways. Microcystins are a chemically diverse family of potent peptide toxins and the end-products of a hybrid NRPS and PKS secondary metabolic pathway. They are produced by a variety of cyanobacteria and are responsible for the poisoning of humans as well as the deaths of wild and domestic animals around the world. The chemical diversity of the microcystin family is attributed to a number of genetic events that have resulted in the diversification of the pathway for microcystin assembly.

**Results:**

Here, we show that independent evolutionary events affecting the substrate specificity of the microcystin biosynthetic pathway have resulted in convergence on a rare [*D*-Leu^1^] microcystin-LR chemical variant. We detected this rare microcystin variant from strains of the distantly related genera *Microcystis*, *Nostoc*, and *Phormidium*. Phylogenetic analysis performed using sequences of the catalytic domains within the *mcy* gene cluster demonstrated a clear recombination pattern in the adenylation domain phylogenetic tree. We found evidence for conversion of the gene encoding the McyA_2_ adenylation domain in strains of the genera *Nostoc* and *Phormidium*. However, point mutations affecting the substrate-binding sequence motifs of the McyA_2_ adenylation domain were associated with the change in substrate specificity in two strains of *Microcystis*. In addition to the main [*D*-Leu^1^] microcystin-LR variant, these two strains produced a new microcystin that was identified as [Met^1^] microcystin-LR.

**Conclusions:**

Phylogenetic analysis demonstrated that both point mutations and gene conversion result in functional *mcy* gene clusters that produce the same rare [*D*-Leu^1^] variant of microcystin in strains of the genera *Microcystis*, *Nostoc*, and *Phormidium*. Engineering pathways to produce recombinant non-ribosomal peptides could provide new natural products or increase the activity of known compounds. Our results suggest that the replacement of entire adenylation domains could be a more successful strategy to obtain higher specificity in the modification of the non-ribosomal peptides than point mutations.

## Background

Non-ribosomal peptides are an important class of secondary metabolites produced by a range of bacteria and fungi [1-3]. These peptides have many biotechnological and pharmaceutical applications such as the antibiotics penicillin [4] and daptomycin [5], and the anticancer bleomycin [6]. They are synthesized on large modular non-ribosomal peptide synthetase (NRPS) and polyketide synthase (PKS) enzyme complexes. Each NRPS module is responsible for the recognition and incorporation of an amino acid during elongation of the peptide intermediate. The basic NRPS module has a condensation (C), adenylation (A), and peptidyl carrier protein (PCP) domains. The adenylation domain is responsible for the selection and activation of amino acids in the form of aminoacyl adenylates [7]. It is followed by the peptidyl carrier protein and condensation domains, the former holding the activated amino acid and the latter making a peptide bond between two adjacent amino acids [2]. In addition, auxiliary enzymes may be present and have activities such as the epimerization, cyclisation, *N*-methylation, formylation, and reduction of amino acids [1,3]. The large variety of non-proteinogenic amino acids and hydroxyl acids that can be incorporated and further modified by tailoring enzymes allows the production of highly complex peptides.

**Figure 1:**
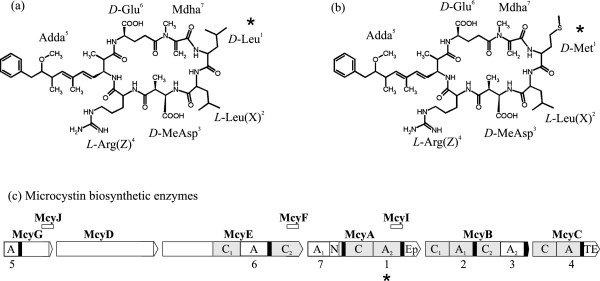
**Microcystin chemical structures and biosynthetic enzymes.** (**a**) [*D*-Leu^1^] microcystin-LR and (**b**) [*D*-Met^1^] microcystin-LR. MeAsp: D-erythro-β-methylaspartic acid. Adda: (2S,3S,8S,9S)-3-,amino-9-methoxy-2,6,8-trimethyl-10-phenyldeca-4,6-dienoic acid. Mdha: N-methyldehydroalanine. X and Z are the highly variable positions. (**c**) NRPS and PKS involved in microcystin synthesis from *Microcystis*. The order of the enzymes corresponds to the assembly of microcystin (each position of the microcystin structure is indicated under each adenylation domain). N. *N*-methyltransferase, Ep. epimerization, TE. thioesterase domains are shown in white and the PCP. peptidyl carrier protein domain in black. Regions included in the phylogenetic analysis are indicated in grey, in addition of their respective PCP domains.

Microcystins are the most frequently reported cyanobacterial toxins in aquatic blooms. They are small cyclic heptapeptides with extensive variation in amino acid residue composition and are commonly produced by planktonic strains in fresh and brackish water [8]. Microcystins are potent inhibitors of serine/threonine protein phosphatases (PP1 and PP2A) and can cause human intoxication, tumor promotion, and death [9]. The general structure of microcystin is cyclo(−*D*-Ala^1^-*X*^2^-*D*-MeAsp^3^-Z^4^-Adda^5^-*D*-Glu^6^-Mdha^7^-) (Figure 1a and b). A hybrid NRPS-PKS enzyme complex directs the synthesis of microcystin and is encoded in the 55–55.6 kb (*mcy*) gene cluster (Figure 1c) [10-13].

Phylogenetic studies indicate that the *mcy* gene cluster has an ancient origin among cyanobacteria [14-17]. Other studies suggest that horizontal transfer, gene loss, and recombination events in the microcystin gene cluster explain the distribution and variation of the genes among the closely related *Microcystis* spp. [18-20]. Recombination events affecting the adenylation domain have been described in genes encoding McyA_1_, McyB_1_ and McyC [16,18,19,21-25]. Positive selection acting on the adenylation domains of McyB_1_ and McyC was reported as the possible cause of the large number of microcystin variants produced by cyanobacteria [24]. Deletion of the entire *N*-methyltransferase domain of *mcyA* in *Anabaena* or point mutations in this gene in *Microcystis* were associated with the absence of *N*-methylation in the microcystins produced by these strains [23,26]. Furthermore, recombination in the same region (*mcyA*_*1*_) was related to the synthesis of microcystins containing 2-amino-2-butenoic acid (Dhb) in some strains of the genus *Planktothrix*[25]. Such genetic rearrangements, positive selection, and recombination events act to increase the chemical variability of microcystins found in nature. However, the production of the same rare microcystin variant in disparate taxonomic lineages of cyanobacteria raises questions about the genetic mechanism underlying this phenomenon [27-31]. Evolutionary diversification of NRPS and PKS pathways is achieved through genetic mechanisms such as recombination, duplication, fusion or fission of genes, deletion or substitutions of domains, circular permutations, gene loss and horizontal gene transfer [32,33]. Here, we show that the production of a rare [*D*-Leu^1^] microcystin (MC) variant in three distantly related genera of cyanobacteria is the result of three independent evolutionary events leading to convergence on the same chemical structure. There is an interest in engineering non-ribosomal peptide biosynthetic pathways in order to increase the production levels of known compounds or create new bioactive compounds [3]. Our results suggest that the replacement of entire adenylation domains might be a more successful strategy for changing substrate specificity in the engineering of NRPS than point mutations.

## Results

### Production of the [*D*-Leu^1^]MC variant by taxonomically disparate cyanobacteria

**Figure 2:**
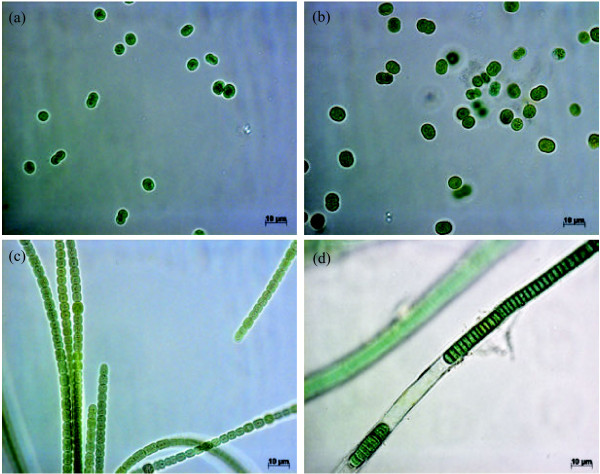
**Photomicrographs of the studied strains.** (**a**) *Microcystis aeruginosa* NPLJ-4. (**b**) *Microcystis* sp. RST 9501. (**c**) *Nostoc* sp. UK89IIa. (**d**) *Phormidium* sp. CENA270.

**Figure 3:**
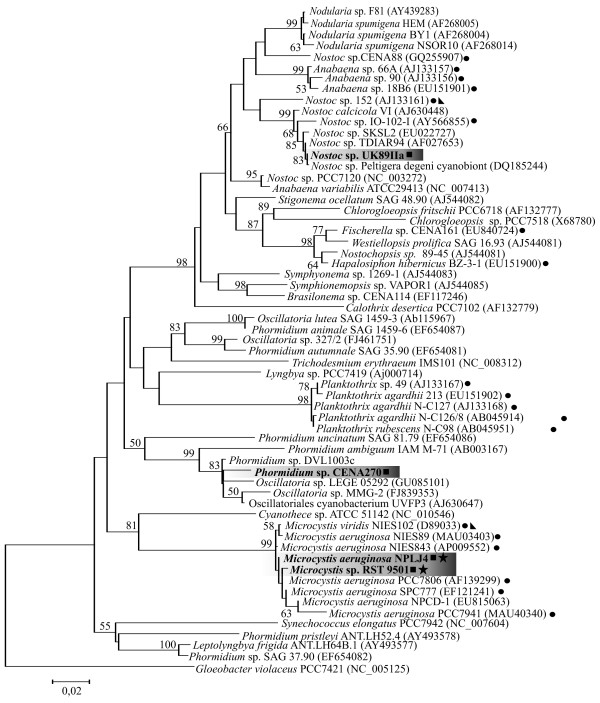
**Phylogenetic analysis of the 16S rRNA gene.** Maximum-likelihood tree based on the 16S rRNA gene. Bootstrap values above 50% from 1000 maximum-likelihood bootstrap replicates are given at the nodes. The studied strains are in bold and highlighted. Symbols for microcystin variants in position one: ● [*D*-Ala^1^]; ■ [*D*-Leu^1^]; ★ [*D*-Met^1^]; ◣ [*D*-Ser^1^].

We documented the production of the rare [*D*-Leu^1^]MC variant in morphologically disparate cyanobacteria (Figure 2). These cyanobacteria were assigned to the genera *Nostoc*, *Phormidium* and *Microcystis*. *Nostoc* sp. strain UK89IIa was identified based on morphology and 16S rRNA gene sequence similarity (Figures 2 and 3). The *Phormidium* sp. CENA270 strain has a thick sheath, tangled filaments and small (≤6.3 μm) cells that indicate the presence of similar characters with *Phormidium*, even though the cells in the trichomes are distinctly wider (5–6.3 μm) than their length (1–3 μm). The 16S rRNA gene sequence of strain CENA270 was 98% of *Phormidium* sp. DVL1003c, which also produces microcystin. These two strains form a clade with *Phormidium*, *Lyngbya*, *Oscillatoria* and Oscillatoriales strains in the 16S rRNA trees (Figure 3; Additional file 1: Figure S1). The two *Microcystis* strains had previously been identified [27,34].

*Nostoc* sp. UK89IIa produced four variants of microcystins, while *Phormidium* sp. CENA270 produced five variants (Table 1). All of the detected microcystin variants produced by these two strains contained *D*-Leu, and none of the strains produced detectable levels of microcystins that contained *D*-Ala. The two *Microcystis* strains produced at least twelve variants altogether (Table 1, Additional file 1: Table S2 and Additional file 2: Table S1). However, 97% of the microcystin variants in *Microcystis aeruginosa* NPLJ-4 and 80% of the microcystin produced by *Microcystis* sp. RST 9501 contained *D*-Leu at position 1 (Table 1, Additional file 2: Table S1). We carried out further chemical analysis to characterize the new microcystin variants produced by these strains.

**Table 1:** Chemical variants of microcystin detected in LC-MS/MS analyses

**Microcystin variant**	**[M+H]**^**+ **^**(*m/z)***	***Microcystis aeruginosa *NPLJ-4**	***Microcystis *sp. RST9501**	***Nostoc *sp. UK89IIa**	***Phormidium *sp. CENA270**
[Leu^1^]MC-LR	1037	76	76	96	31
[Leu^1^, Asp^3^]MC-LR	1023	19	<1	2	18
[Met^1^]MC-LR	1055	1	18	-	-
[Met^1^, Asp^3^]MC-LR	1041	2	-	-	-
[Leu^1^, Dha^7^]MC-LR	1023	1	-	1	-
[Leu^1^]MC-HilR	1051	-	3	-	-
[Leu^1^]MC-HphR	1085	-	-	2	-
[Leu^1^]MC-LHar	1051	-	-	-	7
[Leu^1^]MC-RR	1080	-	-	-	31
[Leu^1^, Asp^3^]MC-RR	1066	-	-	-	13

Values given as the percentage of relative area of the peaks in the protonated molecular ion chromatograms for the main microcystins variants produced by the studied strain. Hil, homoisoleucine. Hph, homophenylalanine. Har, homoarginine.

In all known microcystin variants, the amino acid configuration is *D* in position 1 and *L* in positions 2 and 4 (Figure 1a and b). Therefore, the chirality of the amino acids in positions 1, 2 and 4 of the major microcystin variants produced by the studied strain was investigated by using deuterated acid hydrolysis reagents (DCl, D_2_O). This confirmed that the hydrolyzed microcystins contained *D*-Leu (Additional file 1: Table S2).

### Production of [Met^1^]MC-LR by *Microcystis* spp

Mass spectrometry strongly suggested that *Microcystis* strains NPLJ-4 and RST 9501 produced new microcystin variants that contained Met instead of *D*-Leu (Table 1, Figure 1b). In order to confirm these results, *Microcystis* sp. RST 9501 cells were grown with ^32^S and ^34^S as the sole source of sulfur. LC-MS revealed an increase in [Met^1^]MC-LR protonated ion mass from *m/z* 1,055.5 (control containing ^32^S) to 1,057.5 (^34^S-labelled samples), indicating the presence of a sulfur atom in the microcystin, and the same differences could be visualized in the fragmentation analysis (Additional file 1: Figure S2). The product ion spectra of [Met^1^]MC-LR could be compared with the spectra of [*D*-Leu^1^]MC-LR and -RR (Additional file 1: Figures S2a and S3a and b). The results confirmed the presence of a new variant of [Met^1^]MC in the two *Microcystis* strains NPLJ-4 and RST 9501. However, *Microcystis* sp. RST 9501 produced 14 times more [Met^1^]MC-LR than strain NPLJ-4. On the other hand, *Microcystis aeruginosa* NPLJ-4 produced a larger diversity of microcystin variants, but most of them in trace amounts, such as [Val^1^]MC-LR and [Phe^1^]MC-LR.

### Phylogenetic analysis of microcystin catalytic domains

**Figure 4:**
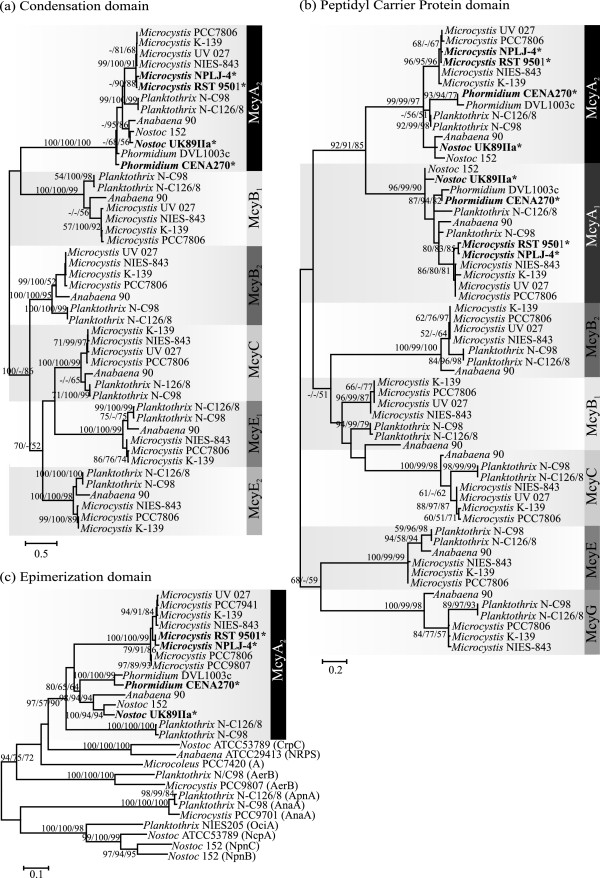
**Conservative evolutionary history of domains surrounding the McyA**_**2**_**adenylation domain in the microcystin biosynthetic gene cluster.** Maximum-likelihood tree based on amino acid sequences of the (**a**) condensation domain, (**b**) peptidyl carrier protein domain and (**c**) epimerization domain within the *mcy* gene cluster. Phylogenetic tree inferred using MEGA 5. Bootstrap values above 50 per cent from 1000 respectively neighbor-joining, maximum parsimony and maximum-likelihood bootstrap replicates are given at the nodes. The studied strains are in bold and indicated with *.

**Figure 5:**
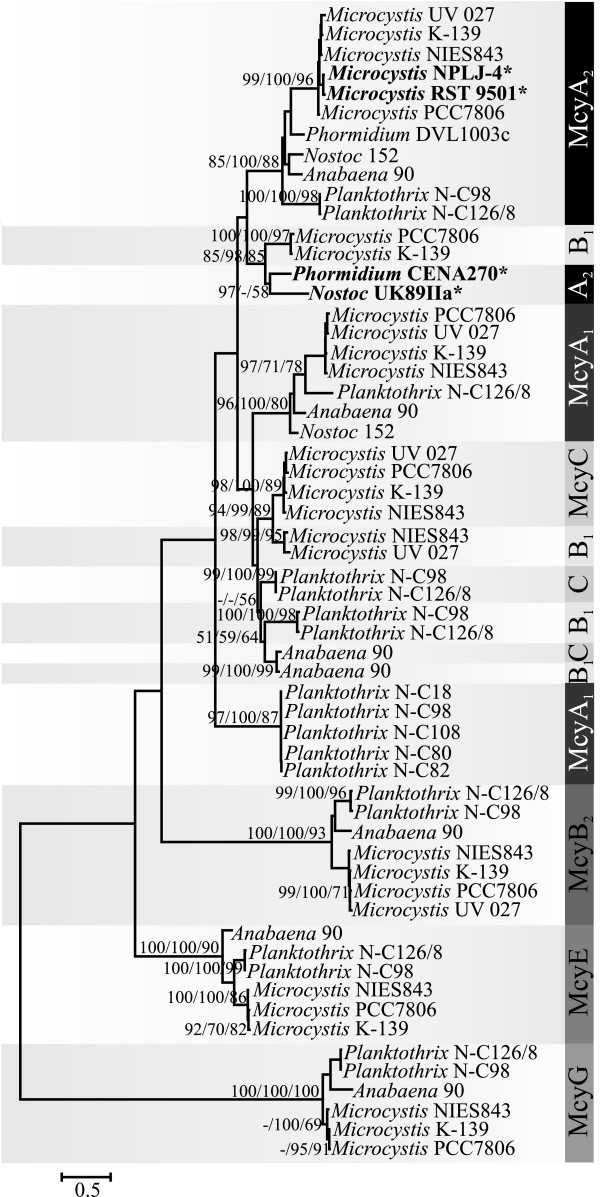
**Evolutionary history of adenylation domains of microcystin biosynthetic gene cluster.** Maximum-likelihood tree based on amino acids sequences of adenylation domain within *mcy* gene cluster. Phylogenetic tree inferred using MEGA 5. Bootstrap values above 50 per cent from 1000 respectively neighbor-joining, maximum parsimony and maximum-likelihood bootstrap replicates are given at the nodes. The studied strains are in bold and indicated with *.

**Figure 6:**
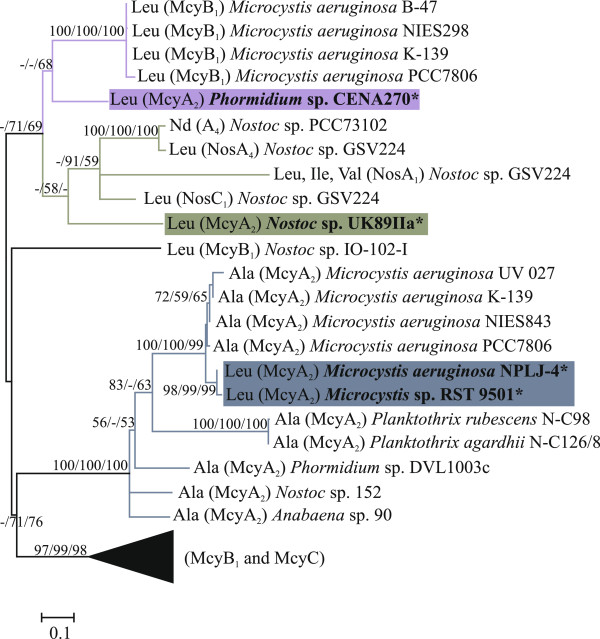
**Independent evolutionary history of McyA**_**2 **_**adenylation domain.** Maximum-likelihood tree based on amino acids sequences inferred using MEGA 5. Bootstrap values above 50 per cent from 1000 respectively neighbor-joining, maximum parsimony and maximum-likelihood bootstrap replicates are given at the nodes. The studied strains are in bold and indicated with *.

In order to understand the order and timing of genetic events leading to the production of the rare [*D*-Leu^1^]MC variant, we conducted phylogenetic analysis of the *mcy* gene cluster. Phylogenetic trees based on concatenated *mcyD* and *mcyE* gene sequences and the housekeeping 16S rRNA gene were robust and found to have a similar pattern (Additional file 1: Figure S4). Strains producing the [*D*-Leu^1^]MC variant do not group together but instead group with strains that produce the [*D*-Ala^1^]MC variant in both trees (Additional file 1: Figure S4). We constructed alignments based on the NRPS catalytic domains encoded in each of the microcystin biosynthetic genes (Figures 1c, 4 and 5). The condensation domains and peptidyl carrier proteins of each module encoded in the *mcy* gene cluster grouped together according to their encoding gene and were placed in separate clades in the phylogenetic tree (Figure 4a and b). A similar pattern was also observed for the adenylation domains of McyG, McyE, and McyB_2_ (Figure 5), and the epimerization domains of McyA (Figure 4c). However, the adenylation domain sequences of McyA_1_, McyA_2_, McyB_1_, and McyC are mixed and do not form separate clades (Figure 5). A phylogenetic tree was constructed using adenylation domain sequences of McyA_2_ and other NRPSs obtained from BLASTp searches of the *nr* database at NCBI (Figure 6). The McyA_2_ adenylation domains of the *Microcystis* strains were grouped in a single well-supported clade, irrespective of whether they produced [*D*-Leu^1^]MCs or [*D*-Ala^1^]MCs variants. By contrast, the McyA_2_ adenylation domain of *Phormidium* sp. CENA270 and *Nostoc* sp. UK89IIa did not group together with other McyA_2_ adenylation domains. Instead, the *Phormidium* sp. CENA270 McyA_2_ adenylation domain was placed in the same clade of McyB_1_ adenylation domain of *Microcystis* strains. The McyA_2_ adenylation domain of *Nostoc* sp. UK89IIa grouped with adenylation domain sequences from *Nostoc punctiforme* PCC73102 and *Nostoc* sp. GSV224.

### Substrate specificity of the McyA_2_ adenylation domain

Conservation of the tertiary structure of adenylation domains makes it possible to predict amino acid binding pockets and consequently the substrate specificity. All strains that produce the [*D*-Ala^1^]MC variants analyzed in this study have identical predicted McyA_2_ adenylation domain binding pocket sequences, with the exception of *Planktothrix agardhii* NIVA-CYA 126/8 (Table 2). There are only two conserved residues (D235 and K517) in the predicted binding pocket in strains producing [*D*-Ala^1^] or [*D*-Leu^1^]MC variants. However, *Nostoc* sp. UK89IIa and *Phormidium* sp. CENA270 have identical predicted binding pockets, differing substantially from those of strains producing [*D*-Ala^1^]MC variants (Table 2). *Microcystis aeruginosa* NPLJ-4 and *Microcystis* sp. RST 9501 have identical predicted binding pockets, which differ by three amino acids at positions 301, 330, and 331 in comparison to strains that activate *L*-Ala (Table 2). Strains producing [*D*-Ala^1^]MC variants have polar amino acids (Thr or Ser) at position 330 of the binding pocket, while strains producing [*D*-Leu^1^]MC variants have hydrophobic non-polar amino acids (Ile and Val) at this position (Table 2).

**Table 2:** **Predicted binding pockets of the adenylation domain of McyA**_2_

**Strain**	**Binding pocket**										**Score %**_**(a)**_	**Predicted**_**(a)**_	**Activated**
	**235**	**236**	**239**	**278**	**299**	**301**	**322**	**330**	**331**	**517**			
*Anabaena* sp. 90	D	L	F	N	N	A	L	T	Y	K	100	Ala	Ala
*Microcystis aeruginosa* NIES-843	-	-	-	-	-	-	-	-	-	-	100	Ala	Ala
*Microcystis aeruginosa* PCC7806	-	-	-	-	-	-	-	-	-	-	100	Ala	Ala
*Microcystis aeruginosa* UV027	-	-	-	-	-	-	-	-	-	-	100	Ala	Ala
*Microcystis aeruginosa* K-139	-	-	-	-	-	-	-	-	-	-	100	Ala	Ala
*Nostoc* sp. 152	-	-	-	-	-	-	-	-	-	-	100	Ala	Ala*
*Phormidium* sp. DVL1003c	-	-	-	-	-	-	-	-	-	-	100	Ala	Ala
*Planktothrix rubescens* NIVA-CYA 98	-	-	-	-	-	-	-	-	-	-	100	Ala	Ala
*Planktothrix agardhii* NIVA-CYA 126/8	-	-	-	-	-	-	-	S	-	-	90	Ala	Ala
*Microcystis* sp. RST 9501	-	-	-	-	-	G	-	I	C	-	70	Cys	Leu, Met
*Microcystis aeruginosa* NPLJ-4	-	-	-	-	-	G	-	I	C	-	70	Cys	Leu, Met ±
*Nostoc* sp. UK89IIa	-	A	W	F	L	G	N	V	V	-	100	Leu	Leu
*Phormidium* sp. CENA270	-	A	W	F	L	G	N	V	V	-	100	Leu	Leu

The adenylation domain of McyA_2_ is responsible for amino acid activation in position one of microcystins.

(a) Prediction obtained using the NRPSpredictor2 program [72,73]. Trace amounts of *Serine and ± Valine or Phenylalanine detected in LC-MS/MS.

### Genetic variations in the McyA_2_ adenylation domain

**Figure 7:**
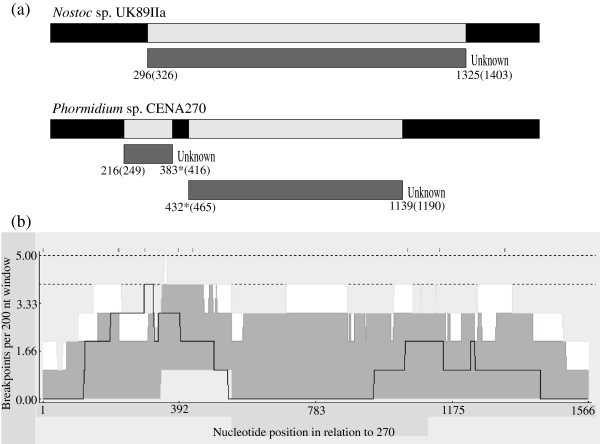
**Recombination breakpoints in the *mcyA***_***2 ***_**gene detected by the RDP3 program.** (**a**) Breakpoint positions detected in three cyanobacterial strains. The position in the alignment is in parentheses. *The breakpoint position is undetermined. (**b**) Potential recombination hotspots within the alignment of *mcyA*_*2*_ genes. The black line corresponds to the breakpoint count within a moving 200-nt-long window. White and grey areas respectively indicate local 99% and 95% confidence intervals of the hot/cold spot test. Upper and lower broken lines respectively indicate 99% and 95% confidence thresholds for the global hotspot test.

*Microcystis* spp. NPLJ-4 and RST 9501, *Nostoc* sp. UK89IIa and *Phormidium* sp. CENA270 produce the same rare [*D*-Leu^1^]MC variant [27,31]. Recombination events affecting the substrate specificity of the *mcyA*_*2*_ gene were detected in *Nostoc* sp. UK89IIa and *Phormidium* sp. CENA270 by four different methods (Table 3). Breakpoints with statistical support within the *mcyA* gene were identified in UK89IIa and CENA270 (Figure 7a). The identified predicted breakpoints were visualized in a recombination breakpoint distribution plot (Figure 7b). A lengthy region replaced in a recombination event in the *mcyA* gene in *Nostoc* sp. UK89IIa (1029 bp) and *Phormidium* sp. CENA270 (167 and 707 bp) was observed in the present study (Figure 7a). The predicted binding pockets responsible for amino acid selection and activation were found within this region in both cases.

**Table 3:** **Recombination events affecting the substrate specificity of the adenylation domain of the*mcyA***_2_**gene**

**Strain**	Parents*		**Average P-value**						
	**Major**	**Minor**	**RDP**	**GENECON**	**BootScan**	**MaxChi**	**Chimaera**	**SiScan**	**3Seq**
*Nostoc* sp. UK89IIa	N152	M9501	1.423x10^-9^	5.882x10^-9^	2.306x10^-3^	5.938x10^-12^	7.331x10^-3^	7.789x10^-16^	2.192x10^-24^
*Phormidium* sp. CENA270	±P1003c	±Ana90	1.242x10^-6^	1.793x10^-18^	3.361x10^-6^	6.848x10^-14^	1.200x10^-3^	7.894x10^-27^	2.410x10^-105^
	¦P1003c	¦M027	4.209x10^-3^	-	-	2.274x10^-5^	9.115x10^-4^	6.746x10^-12^	-

Analysis was performed in the RDP3 program and breakpoint positions are indicated in Figure 7a. The P-value cutoff was chosen as 0.05 and the best signals for recombination are associated with the lowest P-values.

* M7806 = *Microcystis aeruginosa* PCC7806; M139 = *Microcystis aeruginosa* K-139; N152 = *Nostoc* sp. 152; M9501 = *Microcystis* sp. RST 9501; P1003e = *Phormidium* sp. DVL1003c; Ana90 = *Anabaena* sp. 90; M027 = *Microcystis aeruginosa* UV027. Breakpoint positions: ± 432 (undetermined) to 1139 and ¦ 216 to 383 (undetermined).

No evidence for recombination was detected in the *mcyA*_*2*_ gene from *Microcystis aeruginosa* NPLJ-4 or *Microcystis* sp. RST 9501. However, the McyA_2_ adenylation domain of *Microcystis* spp. NPLJ-4 and RST 9501 differed by 17 amino acids residues compared to other McyA_2_ adenylation domains of *Microcystis* strains that produce microcystins containing *D*-Ala (Additional file 1: Figure S5). Five of the 17 amino acids residues were predicted to be located within 8 Å of the substrate, and three of them were considered to be within the binding pocket. Mutants based on *Microcystis* sp. RST 9501 McyA_2_ adenylation domain were constructed to analyze the effect of these three amino acids residues on binding of the predicted substrate amino acids. Surprisingly, the results obtained in the ATP-pyrophosphate exchange assay showed the highest specificity for *L*-Val in all the mutants and the wild type (Additional file 1: Figure S6). Furthermore, the results indicated a higher activation of *L*-Ile instead of the expected *L*-Leu and *L*-Ala. The high levels of miscognate activation indicate that the catalytic efficiency of the enzymes in recognizing nonpolar amino acid was generally higher, although some specificity was observed for *L*-Tyr (Additional file 1: Figure S6).

## Discussion

### Identification of the rare [*D*-Leu^1^]MC and other microcystin variants

Microcystins form a large family of cyclic toxins characterized by a highly conserved chemical structure with an extensive amino acid composition at the two variable positions, two and four (Additional file 1: Figure S7 and Table S3). In this study, we detected rare [*D*-Leu^1^]MC variants from strains of the distantly related genera *Microcystis*, *Nostoc*, and *Phormidium*. Almost all microcystins reported in the literature contain *D*-Ala in position 1 [8]. Previously, microcystins containing *D*-Leu [27-31], Gly [35], and *D*-Ser [36,37] have also been reported. The [*D*-Leu^1^]MC variant has previously been found from *Microcystis aeruginosa* NPLJ-4, *Microcystis* sp. RST 9501, and water blooms from Brazil and Canada dominated by *Microcystis* strains [27-31,34]. The production of microcystins in lichen thalli by *Nostoc* symbionts has previously been reported [37,38]. *Nostoc* strains isolated from lichen symbiosis produce a large variety of microcystins including the [*D*-Leu^1^]MC variant [31].

Microcystins are best known from aquatic habitats, where they are frequently reported from blooms. Although microcystins are more commonly detected in planktonic strains, terrestrial and benthic strains have also been reported to be producers of these compounds [8,39]. Benthic environmental samples containing microcystins have been reported from Switzerland [40], Spain [41], Australia [42], and Antarctica [43]. Microcystin production in isolated cyanobacterial strains from benthic environments has been reported from Egypt [44], New Zealand [45], and the USA [46]. It is not always clear which cyanobacterium produces the toxin in benthic mats of cyanobacteria. A strain of the genus *Phormidium* was isolated from the walls of a reservoir in the USA and shown to produce a range of microcystin variants, all of which contained *D*-Ala (Additional file 1: Table S3) [46]. Our results demonstrated that *Phormidium* sp. CENA 270, isolated from a pond in the northeast of Brazil, also produces microcystins but with *D*-Leu in place of *D*-Ala. In the phylogenetic analysis of the 16S rRNA gene, the two *Phormidium* strains cluster together with *Lyngbya*, *Oscillatoria*, *Phormidium* and Oscillatoriales (Additional file 1: Figure S1). The biomass of benthic strains can go unnoticed to the casual observer but be massive enough to cause animal poisonings [40,45]. Moreover, when the strains lyse, microcystins are released into the water, which suggests that the analysis of toxic benthic cyanobacteria is also important in water-quality management.

The chemical structure of microcystins is highly conserved, with variation at X and Z positions (Figure 1a and b) resulting in over 86 reported variants [39]. In this study, we demonstrated that *Microcystis* strains NPLJ-4 and RST 9501 produce new microcystin variants containing methionine in addition to the rare [*D*-Leu^1^]MC variants. Met is also present in oscillamide B [47] and in microcystin-M(O)R and -YM [48]. *Nodularia spumigena* strains also produce nodulapeptins, which commonly contain Met [49,50]. According to analysis using the NRPS Norine database, Met contains a methylthiol group and is rare in non-ribosomal peptides [51]. The presence of the highly active sulfhydryl group in the thiol group could explain the scarcity of secondary metabolites containing Met or Cys. If amino acid recognition by the McyA_2_ adenylation domain is not strict, the incorporation of Met instead of Leu is logical because of the similar size and hydrophobicity of the side chains. Here, we demonstrated that methionine is incorporated in the microcystins produced by *Microcystis* strains from brackish water. Microcystin variants are constantly being discovered, making the microcystin family extremely diverse, and posing a challenge for the detection of microcystins from water samples.

### Convergence on [*D*-Leu^1^]MC variant chemical structure

Phylogenetic analysis of the McyA_2_ adenylation domains provided evidence for independent evolutionary events affecting the substrate specificity of the enzyme in three disparate genera of cyanobacteria (Figures 5 and 6). Breakpoint analysis suggests the replacement of almost the entire substrate specificity-conferring portion of the adenylation domain in *Phormidium* sp. CENA270 and *Nostoc* sp. UK89IIa. These gene conversions dramatically altered the predicted substrate specificity of the McyA_2_ adenylation domain in these strains and are linked to the synthesis of the [*D*-Leu^1^]MC variant. However, point mutations affecting the substrate specificity of the McyA_2_ adenylation domain in *Microcystis* strains NPLJ-4 and RST 9501 led to the synthesis of the [*D*-Leu^1^]MC and [Met^1^]MC variants.

*Phormidium* sp. CENA270 and *Nostoc* sp. UK89IIa are not grouped together with other McyA_2_ adenylation domains (Figure 6). The McyA_2_ adenylation domain of *Phormidium* sp. CENA270 grouped with the McyB_1_ adenylation domain from *Microcystis* strains, which produce microcystin variants containing *D*-Ala [10,52]. The adenylation domains of NosA_1_ and NosC_1_ from the nostopeptolide gene cluster are placed in the same clade with the McyA_2_ adenylation domain from *Nostoc* sp. UK89IIa (Figure 6). They are involved in the incorporation of Ile/Leu/Val and Leu, respectively, in *Nostoc* sp. GSV224 [53].

Genetic variation in the microcystin synthetases can be visualized in the phylogenetic trees showing two different patterns. While the amino acids of condensation, peptidyl carrier protein, and epimerization domain regions can be grouped according to the enzyme sequence (McyA, McyB, McyC, McyE, and McyG grouped together), the adenylation domain phylogeny clearly indicates recombination (Figures 4 and 5). Recombination in adenylation domains has previously been described for the adenylation and condensation domains of McyB_1_ and McyC [16]. The recombination and positive selection in the McyB_1_ adenylation domain are involved in the high variability of amino acids incorporated at position 2 of the microcystin [16,18,22,24,25]. These genetic events have been related to the increase in the chemical diversity of microcystin. Interestingly, our results show that these different evolutionary events are involved in the convergence of the [*D*-Leu^1^]MC-LR.

Nevertheless, the selective forces behind this convergent evolution remain unclear. Competition in brackish water and different seasonal periods have possibly acted as selective forces. The chemical diversity of microcystins could be related to protein phosphatase inhibition as a form of chemical defense, for example against predators. Previous studies have indicated that microcystins can affect some predators, acting as metal chelators, in gene regulation, or in the inter- and intra-specific signaling [54-59]. However, microcystins join a large number of secondary metabolites produced by different organisms that have no assigned biological function. According to the most accepted view, these compounds are produced due their ecological or physiological function and benefits for the producer organisms [60]. However, more information is still needed concerning the advantages in the production of these secondary metabolites. The biological role of a mixture of different bioactive compounds produced by the same strain would be interesting to study.

### Prediction of McyA_2_ adenylation domain substrate specificity

The eight to ten amino acid residues forming the adenylation domain binding pocket are the main determinants of substrate specificity [7,61,62]. In our study, *Phormidium* sp. CENA270 and *Nostoc* sp. UK89IIa were shown to produce [*D*-Leu^1^]MC variants and have identical binding pocket sequences (Table 2). Such amino acids signatures had been already described as presenting Leu specificity [7]. The *Microcystis* strains NPLJ-4 and RST 9501 differ in the binding pocket positions 301, 330, and 331 from the strains producing [*D*-Ala^1^]MCs. Residues at positions 301 and 330 are regarded to be less variable than at position 331 [7].

The adenylation domain binding pocket of *Microcystis* strains NPLJ-4 and RST 9501 has three different amino acid residues and a broader diversity of microcystin variants at position 1. Despite the fact that almost the entire binding pocket of *Phormidium* sp. CENA270 and *Nostoc* sp. UK89IIa differs from the other studied strains, only microcystin variants containing Leu at position 1 were detected. Re-engineering of non-ribosomal peptides has been a challenge in order to synthesize new peptides or to increase the activity of known compounds. The engineering of NRPSs to change substrate specificity can in some cases be achieved by point mutations. However, our results suggest that the replacement of entire domains might be a more successful strategy for producing a single product.

Replacement of almost the entire McyA_2_ adenylation domain in *Nostoc* sp. UK89IIa and *Phormidium* sp. CENA270 resulted in specificity towards Leu. Neither strain produced detectable levels of microcystin variants that contain other amino acids at this position. The recombination detected in the *mcyA*_*2*_ gene of these strains affects the substrate-conferring portion of the McyA_2_ adenylation domain, which is important for the selection and activation of amino acids [7,61,62]. Previously, it has been reported that recombination among different adenylation domains from *mcyB1* and *mcyC* genes has led to a change in amino acid activation [24].

We designed an experiment in order to test whether point mutations at positions 301, 330, and 331 could change the substrate specificity of the adenylation domain. However, single amino acids changes did not have the expected results. All the constructs and the wild type were found to activate valine in ATP-pyrophosphate (PPi) exchange assays. A previous study [7] demonstrated that in the case of single or multiple mutations, the specificity of the wild type is not lost, but there is an increase in new substrate specificity. A comparison of adenylation domains from *Microcystis* strains that activate Ala and Leu reveals that several amino acid residues differ between them (Additional file 1: Figure S5). Of these different amino acid residues, five are 8 Å or less distant from the substrate and only three belong to the binding pocket. Although it is predicted that amino acid residues in the binding pocket are involved in selectivity, the catalytic efficiency could also be affected by the tertiary structure and proteinogenic surrounding area of the adenylation domain [63]. Promiscuity of the enzymes, allowing them to activate different substrates, could also be involved in the high variability of microcystin variants. Promiscuous activation of amino acids with a hydrophobic side chain by TycA, involved in the synthesis of the antibiotic tyrocidine A, has been reported [64]. Moreover, adenylation domains activating multiple substrates have been described from the fengycin [65], lychenysin [66], nostopeptolide [53], and cyanopeptolin [67] biosynthetic pathways.

## Conclusion

Our study revealed that independent gene conversion events and point mutations led to the production of the same microcystin variant by strains belonging to three different cyanobacterial orders. The large chemical diversity of microcystins is proposed to be mostly the result of genetic rearrangements, positive selection, and recombination acting to increase structural diversity. Furthermore, the replacement of the entire adenylation domain seems to result in a more specific change in non-ribosomal peptides than point mutation. New variants of [Met^1^]MCs were detected in *Microcystis* strains NPLJ-4 and RST 9501. Our study also revealed a new cyanobacterial strain (*Phormidium* sp CENA270) producing a rare variant of the potent hepatotoxic microcystin. This finding expands on the recent increase in the detection of microcystin-producing terrestrial and benthic cyanobacterial strains.

## Methods

### Studied strains and culture conditions

*Phormidium* sp. CENA270 was isolated from a pond during the rainy season in Paulista (Paraiba, Brazil) and maintained in BG-11 medium [68], but supplemented with half the described amount of combined nitrogen. *Nostoc* sp. UK89IIa was isolated from the lichen *Peltigera neopolydactyla* sampled in Laukaa (Finland) and maintained in Z8 medium [69] lacking a source of combined nitrogen. *Microcystis* strains NPLJ-4 and RST 9501 were respectively isolated from the brackish water of Jacarepaguá Lagoon, Rio de Janeiro [34], and the Patos Lagoon estuary, Rio Grande do Sul [27], in Brazil and maintained in Z8 medium [69] with and without a source of combined nitrogen. The biomass for chemical and molecular analysis was obtained by growing the strains in 2 x 3 L of culture medium under constant light of 3–8 μmol m^-2^ s^-1^ and a temperature of 24 ± 1°C. *Microcystis* sp. RST 9501 was grown in Z8 medium replaced with stable isotope ^34^S-labeled MgSO_4_.7H_2_O (catalogue no. IS7080; 90 atom % ^34^S; Icon) for the detection and identification of sulfur-containing microcystins.

### Sequencing and phylogenetic analysis

Freeze-dried cells (6–50 mg) were used for DNA extraction with a DNeasy Plant mini kit (Qiagen). Two different sizes of glass beads (180 μm and 425–600 μm, Sigma-Aldrich) were added and the cells were disrupted by shaking at 6.5 m s^-1^ for 60 seconds in 3 batches using a FastPrep (M.P. Biomedicals). An additional 1 h incubation at 100°C was necessary in order to extract *Phormidium* sp. CENA270 genomic DNA due the thick sheath surrounding the trichomes of this strain. PCR reaction conditions are described in Additional file 3. The PCR products were cloned in pCR®2.1-TOPO (TOPO TA Cloning, Invitrogen) with the following modifications from the manufacturer’s instructions: the entire cloning reaction was used for transformation in 25 μL of TOP10 competent cells, 100 μL of SOC medium was added to the mixture after heat shock and incubated for 20 min in shaker (160 RPM) at 37°C, and the entire reaction volume was plated in LB plates containing 150 μg mL^-1^ of ampicillin.

Plasmid extraction was performed using the QIAprep Spin Miniprep kit (Qiagen) and cycle sequencing was carried out using an ABI Prism® BigDye® Terminator v3.1 cycle sequencing kit. The oligonucleotide primers used to sequence PCR products are given in Additional file 1: Table S4. Sanger sequencing was performed in an ABI PRISM 310 Genetic Analyzer. Contigs were aligned in the program Phred/Phrap/Consed (Philip Green, University of Washington, Seattle, USA), accepting bases with quality >20. BLASTn was used to search the nr database at NCBI for related strains to be included in phylogenetic analysis. GenBank accession numbers are indicated in Additional file 1: Table S5. Maximum-likelihood trees were constructed in MEGA 5.0 [70]. The best substitution model for each sequence was chosen based on analysis in MEGA 5.0 (K2 + G for the small tree of 16S rRNA; GTR + G + I for *mcyD* concatenated with *mcyE*; K2 + G + I for long tree of 16S rRNA; JTT + G + I for condensation, adenylation, and epimerization domains sequences of McyA; and JTT + G for peptidyl carrier protein domain sequences of McyA). Neighbor-joining and maximum parsimony trees were constructed using the respective methods: JTT + G and CNI on random trees. The domains present in the McyA amino acid sequences were detected in the program PKS/NRPS Analysis [71]. Adenylation domain substrate specificity prediction was performed using the program NRPSpredictor2 [72,73].

### Recombination test

The nucleotide sequences of 13 cyanobacterial strains (Additional file 1: Table S5) corresponding to second adenylation domain of *mcyA* (*mcyA*_*2*_) were aligned in ClustalW (MEGA 5.0). The recombination analyses were performed in the program RDP3 [74,75], which implements different methods to detect recombination. The methods used in this study were: original RDP [76], BOOTSCAN [77], GENECONV [78], MAXCHI [79], CHIMAERA [80], SISCAN [81] and 3Seq [82]. Default parameters were used and a P-value cutoff was chosen as 0.05. Nucleotide sequence comparison was performed in BLASTn (NCBI database). A breakpoint distribution was plotted, allowing the visualization of recombination hotspots [74]. The phylogenetic compatibility matrix was calculated by TreeOrder scan in the program SSE [83].

### McyA_2_ adenylation domain mutation and heterologous expression

A fragment containing the *mcyA*_*2*_ adenylation domain was obtained from *Microcystis* sp. RST 9501 by PCR using the primers RSTPETF and RSTPETR (Additional file 1: Table S6). The PCR reaction conditions are described in Additional file 3. The 1,646 bp DNA fragment was cloned in pET101/D-TOPO (Invitrogen) and transformed in TOP10 chemically competent *Escherichia coli* cells. Mutants were obtained by site-directed mutation with PCR mutagenesis using specific primers (Additional file 1: Table S6). Detailed information on mutagenesis is presented in Additional file 3. Mutant G301A contains an Ala instead of Gly at position 301, mutant I330T a Thr instead of Ile at position 330, mutant C331Y a Tyr instead of Cys at position 331, and mutant G301A, I330T, C331Y has all three mutations. The presence of the desired mutation was verified by sequencing. Mutated and wild type adenylation domains were cloned into pFN18A (HaloTag® 7) T7 Flexi® vector (Promega, WI, USA) and transformed in *Escherichia coli* KRX competent cells (Promega) following the manufacturer’s instructions. The adenylation domain was heterologously expressed and purified using the HaloTag® Protein Purification System (Promega). The ATP-pyrophosphate exchange assay was performed as previously described [49].

### Chemical analysis

Identification of microcystin variants was performed using an Agilent 1100 Series LC/MSD Trap XCT Plus high-performance liquid chromatograph mass spectrometer (Agilent Technologies). Freeze-dried biomass was extracted with 1 mL of methanol in a FastPrep homogenizer (M.P. Biomedicals). The supernatant was injected to a Luna C18(2) column (150 × 2.1 mm, 5 μm, Phenomenex) for the LC-MS analysis. Electrospray ionization in positive mode was used and the product ion spectra of protonated microcystins were analyzed to identify the structure of the variant.

The remains of the methanol extracts were mixed with water and dichloromethane in equivalent proportions (1:1:1). The hydrophilic upper phase was diluted with water and passed through a preconditioned SPE column (strata 8B-S100-UBJ, Phenomenex). Microcystin-containing fractions were recovered in 1 mL of methanol. Microcystins were isolated and purified by HPLC (HP 1100 Series modular chromatograph, Agilent Technologies). The microcystin fractions were hydrolyzed with 200 μL of 6 M deuterium chloride (catalog no. 543047; 35 wt % solution in D_2_O, 99 atom % D, Sigma-Aldrich) and prepared for enantiomeric amino acid analysis using FDAA (Pierce) as a Marfey reagent, as described previously [84]. Chiral analysis of the amino acids alanine, leucine, arginine, and homoarginine was carried out using LC-MS. Amino acids from the common [*D*-Ala^1^] MC-LR and -RR variants produced by *Anabaena* sp. 90 were analyzed as references. Detailed information on the chemical analysis is provided in Additional file 3.

## Abbreviations

NRPS: Non-ribosomal peptide synthetase; PKS: Polyketide synthase; NRPS-PKS: Hybrid non-ribosomal peptide synthetase and polyketide synthase; A: Adenylation domain; C: Condensation domain; PCP: Peptidyl carrier protein domains; E: Epimerization domain; MC: Microcystin.

## Competing interests

The authors declare that they have no competing interests.

## Authors’ contributions

All authors contributed to writing of the manuscript and interpretation of the data. TKS, DPF, JJ and MW participated in the design of the experiments. TKS and DPF carried out the molecular experiments and analysis. TKS and LR carried out the biochemical assay. TKS, JJ, UK and MW carried out the chemical experiments and analysis. MFF, JSY and LR revised the manuscript. KS made the study financially feasible and was involved in the design, drafting and revision of the manuscript. All authors read and approved the final manuscript.

Cover page photo: *Peltigera neopolydactyla* (Photo: Jouko Rikkinen); Patos Lagoon estuary, Rio Grande do Sul (Photo: João Sarkis Yunes); and Paulista, Paraiba, Brazil (Photo: João Luiz da Silva).

## Supplementary Material

Additional file 1: Table S2 Chiral analysis of microcystin amino acids. Table S3: Amino acids incorporated by each adenylation domain of microcystin biosynthetic enzymes for the studied strains. Table S4: Primers used in this study. Table S5: Access number of strains compared in this study. Table S6. Primers designed for PCR mutagenesis. Figure S1: Phylogenetic analysis of 16S rRNA gene focusing in *Phormidium* sp. CENA270. Figure S2: Product ion spectra of [Met^1^] MC-LR of *Microcystis* sp. RST 9501 in the labeling experiment. Figure S3: Product ion spectra of protonated [Leu^1^]microcystins of *Phormidium* sp. CENA270. Figure S4. Phylogenetic congruence between housekeeping and microcystin synthetase genes. Figure S5: Comparison of McyA_2_ adenylation domain sequences from *Microcystis* strains. Figure S6: ATP-PPi exchange assay. Figure S7: Relative quantity of amino acids in detected microcystins for strains included in the phylogenetic tree. Click here for file

Additional file 2: Table S1 Assignment of the main ions from microcystin variants produced by the studied strains in LC-MS/MS. Click here for file

Additional file 3 **Supplementary information in Methods.** Additional file 3 is a PDF file that contains further information to complement the Methods sub-sections: Sequencing and phylogenetic analysis; McyA_2_ adenylation domain mutation and heterologous expression; and Chemical analysis. Click here for file
