# Supplementary material for: Convergent evolution of [D-Leucine1] microcystin-LR in taxonomically disparate cyanobacteria
Source: BMC Evol Biol. 2013 Apr 19;13:86. doi: 10.1186/1471-2148-13-86 (PMC3640908; doi:10.1186/1471-2148-13-86)
Supplement: Additional file 1: Table S2 — Chiral analysis of microcystin amino acids. Table S3: Amino acids incorporated by each adenylation domain of microcystin biosynthetic enzymes for the studied strains. Table S4: Primers used in this study. Table S5: Access number of strains compared in this study. Table S6. Primers designed for PCR mutagenesis. Figure S1: Phylogenetic analysis of 16S rRNA gene focusing in Phormidium sp. CENA270. Figure S2: Product ion spectra of [Met1] MC-LR of Microcystis sp. RST 9501 in the labeling experiment. Figure S3: Product ion spectra of protonated [Leu1]microcystins of Phormidium sp. CENA270. Figure S4. Phylogenetic congruence between housekeeping and microcystin synthetase genes. Figure S5: Comparison of McyA2 adenylation domain sequences from Microcystis strains. Figure S6: ATP-PPi exchange assay. Figure S7: Relative quantity of amino acids in detected microcystins for strains included in the phylogenetic tree. [file 1471-2148-13-86-S1.pdf]

Supplementary Materials

Table S2. Chiral analysis of microcystin amino acids. Analysis of the *L*- and *D*- Leucine, *L*-Arginine and *L*-Homoarginine from the peak areas of the protonated amino acid ion chromatograms. Values are relative (%) amounts of L/D-forms of leucine and relative (%) amounts of L-forms of Arg/Har. *D*-Arginine and *D*-Homoarginine were not detected. Retention times (min): *L*-Leu (31.8-31.9), *D*-Leu (35.1-35.2), *L*-Arg (13.9-14.1), and *L*-Har (14.7-14.8). *Anabaena* sp. 90 was included as reference strain.

**Source**	**MC variants**	***L*-Leu**	***D*-Leu**	***L*-Arg**	***L*-Har**
Anabaena sp. 90	[Ala^1]MC-RR	-	-	100	-
	[Ala^1]MC-LR	100	-	100	-
Microcystis aeruginosa NPLJ-4	[Leu^1]MC-LR	43	57	100	-
	[Leu^1,Asp^3]MC-LR	42	58	100	-
Microcystis sp. RST 9501	[Leu^1]MC-LR	46	54	100	-
	[Met^1]MC-LR	100	-	100	-
Nostoc sp. UK89IIa	[Leu^1]MC-LR	42	58	100	-
Phormidium sp. CENA270	[Leu^1]MC-RR	-	100	100	-
	[Leu^1]MC-RHar	-	100	-	100
	[Leu^1]MC-LR	44	56	100	-
	[Leu^1]MC-LHar	48	52	-	100

Table S3. Amino acids incorporated by each adenylation domain of microcystin biosynthetic enzymes for the studied strains.

Strain	Microcystin variants	Amino acids in microcystin structure $^{(position)}$ correspondent to each A domain							%	Ref±
		McyA$_2^1$	McyB$_1^2$	McyB$_2^3$	McyC^4	McyG^5	McyE^6	McyA$_1^7$	MC*	
Anabaena sp. 90	MC-LR	Ala	Leu	MeAsp	Arg	Adda	Glu	Mdha	55	1
	[D-Asp^3]MC-LR	-	-	Asp	-	-	-	-	34	
	MC-RR	-	Arg	-	-	-	-	-	5	
	[D-Asp^3]MC-RR	-	Arg	Asp	-	-	-	-	2	
	MC-HilR	-	Hil	-	-	-	-	-	1.5	
	[D-Asp^3]MC-HilR	-	Hil	Asp	-	-	-	-	<1	
	[MeSer^7]MC-LR	-	-	-	-	-	-	MeSer	<1	
	[DMAdda^5]MC-LR	-	-	-	-	DMAdda	-	-	<1	
	[D-Asp^3, MeSer^7]MC-LR	-	-	Asp	-	-	-	MeSer	<1	
	[Dha^7]MC-LR	-	-	-	-	-	-	Dha	<1	
Anabaena sp. 18B6	[D-Asp^3, Dha^7]MC-RR	-	Arg	Asp	-	-	-	Dha	79	1
	Demethyl- MC-RR	-	Arg	-	-	-	-	-	20	
	[X]MC-RR	-	Arg	-	-	-	-	-	1	
	MC-XR	-	X	-	-	-	-	-	<1	
Anabaena sp. 66A	[Dha^7]MC-HtyR	-	Hty	-	-	-	-	Dha	56	1
	[D-Asp^3, Dha^7]MC-XR	-	X	Asp	-	-	-	Dha	29	
	[L-Ser^7]MC-HtyR	-	Hty	-	-	-	-	L-Ser^7	4	
	[D-Asp^3, L-Ser^7]MC-HtyR	-	Hty	Asp	-	-	-	-	2	
	[Dha^7]MC-LR	-	-	-	-	-	-	Dha	2	
	[Dha^7]MC-HphR	-	Hph	-	-	-	-	Dha	2	
	[D-Asp^3, Dha^7]MC-LR	-	-	Asp	-	-	-	Dha	1	
	[DMAdda^5, (M)dha^7]MC-(H)tyR	-	Hty/Tyr	-	-	DMAdda	-	(M)Dha	<1	
	[D-Asp^3, Dha^7]MC-XR	-	X	Asp	-	-	-	Dha	<1	
	[X]MC-HtyR	-	Hty	-	-	-	-	-	<1	
	[D-Asp^3]MC-XR	-	X	Asp	-	-	-	-	<1	
	[X]MC-(H)tyR	-	Hty/Tyr	-	-	-	-	-	<1	
	MC-XR	-	X	-	-	-	-	-	<1	
	[X]MC-X/HtyR	-	X/Hty	-	-	-	-	-	<1	
	[X]MC-(H)tyR	-	Hty/Tyr	-	-	-	-	-	<1	
	MC-HtyR	-	Hty	-	-	-	-	-	<1	
	[X, L-Ser^7]MC-LR	-	-	-	-	-	-	L-Ser^7	<1	

	[D-Asp[3]]MC-XR	-	X	Asp	-	-	-	-	<1	
	Demethyl-[L-Ser[7]]MC-LR	-	-	-	-	-	-	L-Ser[7]	<1	
	[L-Ser[7]]MC-LR	-	-	-	-	-	-	L-Ser[7]	<1	
	[Dha[7]]MC-FR	-	Phe	-	-	-	-	Dha	<1	
	[D-Asp[3], Dha[7]]MC-FR	-	Phe	Asp	-	-	-	Dha	<1	
	[X]MC-LR	-	-	-	-	-	-	-	<1	
	[D-Asp[3], Dha[7]]MC-HphR	-	Hph	Asp	-	-	-	Dha	<1	
Hapalosiphon hibernicus BZ-3-1	MC-LA	-	-	-	Ala	-	-	-	70	1
	[D-Asp[3]]MC-LA	-	-	Asp	Ala	-	-	-	16	
	MC-RA	-	Arg	-	Ala	-	-	-	7	
	[D-Asp[3]]MC-RA	-	Arg	Asp	Ala	-	-	-	2	
	MC-VA	-	Val	-	Ala	-	-	-	1	
	[D-Asp[3], DMAdda[5]]MC-LA	-	-	Asp	Ala	DMAdda[5]	-	-	1	
	[Dha[7]]MC-LA	-	-	-	Ala	-	-	Dha	1	
	MC-LL	-	-	-	Leu	-	-	-	<1	
	[D-Asp[3]]MC-VA	-	Val	Asp	Ala	-	-	-	<1	
	[D-Asp[3]]MC-LV	-	-	Asp	Val	-	-	-	<1	
	MC-LV	-	-	-	Val	-	-	-	<1	
Microcystis aeruginosa B-47	[Dha[7]]MC-LR	-	-	-	-	-	-	Dha	Ni	2
Microcystis aeruginosa PCC7806	[D-Asp[3]]MC-LR	-	-	Asp	-	-	-	-	52	1
	MC-LR	-	-	-	-	-	-	-	46	
	[MeSer[7]]MC-LR	-	-	-	-	-	-	MeSer	<1	
	[D-Asp[3], Dha[7]]MC-LR	-	-	Asp	-	-	-	Dha	<1	
	[Dha[7]]-MC-LR	-	-	-	-	-	-	Dha	<1	
	[D-Asp[3], MeSer[7]]MC-LR	-	-	Asp	-	-	-	MeSer	<1	
Microcystis aeruginosa K-139	[Dha[7]]MC-LR	-	-	-	-	-	-	Dha	76	3
	[Asp[3], Dha[7]]MC-LR	-	-	Asp	-	-	-	Dha	24	
Microcystis aeruginosa UV027	MC-RR	-	Arg	-	-	-	-	-	100	4
Microcystis aeruginosa NIES298	MC-LR	-	-	-	-	-	-	-	41	11
Microcystis aeruginosa NIES843	MC-RR	-	Arg	-	-	-	-	-	50	5
	MC-LR	-	-	-	-	-	-	-	41	

	MC-YR	-	Tyr	-	-	-	-	-	10	
Microcystis aeruginosa NPLJ-4	[Leu^{1}]MC-LR	Leu	-	-	-	-	-	-	76	6
	[Leu^{1}, Asp^{3}]MC-LR	Leu	-	Asp	-	-	-	-	19	
	[Met^{1}, Asp^{3}]MC-LR	Met	-	Asp	-	-	-	-	2	
	[Met^{1}]MC-LR	Met	-	-	-	-	-	-	1	
	[Leu^{1}, Dha^{7}]MC-LR	Leu	-	-	-	-	-	Dha	1	
	[Phe^{1}]MC-LR	Phe	-	-	-	-	-	-	<1	
	[Val^{1}]MC-LR	Val	-	-	-	-	-	-	<1	
	[Leu^{1}, DMAdda^{5}]MC-LR	Leu	-	-	-	DMAdda	-	-	<1	
Microcystis sp. RST 9501	[Leu^{1}]MC-LR	Leu	-	-	-	-	-	-	76	6
	[Met^{1}]MC-LR	Met	-	-	-	-	-	-	18	
	[Leu^{1}]MC-HilR	Leu	Hil	-	-	-	-	-	3	
	[Leu^{1},Asp^{3}]MC-LR	Leu	-	Asp	-	-	-	-	<1	
Microcystis viridis NIES102	MC-HilR	-	Hil	-	-	-	-	-	19	1
	[Adda/DMAdda^{5}]MC-LR	-	Leu/Hil	-	-	(DM)Adda	-	-	15	
	MC-RR	-	Arg	-	-	-	-	-	10	
	MC-YR	-	Tyr	-	-	-	-	-	9	
	[X, D-Asp^{3}]MC-HilR	-	Hil	Asp	-	-	-	-	6	
	MC-WR	-	W	-	-	-	-	-	5	
	[D-Asp^{3}]MC-LR	-	-	Asp	-	-	-	-	2	
	[D-Asp^{3}]MC-YR	-	Tyr	Asp	-	-	-	-	2	
	MC-FR	-	F	-	-	-	-	-	2	
	MC-HtyR	-	Hty	-	-	-	-	-	2	
	MC-XR	-	X	-	-	-	-	-	2	
	MC-XR	-	X	-	-	-	-	-	2	
	MC-XR	-	X	-	-	-	-	-	1	
	[D-Asp^{3}]MC-WR	-	W	Asp	-	-	-	-	<1	
	[D-Asp^{3}]MC-RR	-	Arg	Asp	-	-	-	-	<1	
	[D-Asp^{3}]MC-XR	-	X	Asp	-	-	-	-	<1	
	[Ser^{1}, D-Asp^{3}, Dha^{7}]MC-LR	Ser	-	Asp	-	-	-	Dha	<1	
	[X, MeSer^{7}]MC-(Hil)R	-	Hil	-	-	-	-	MeSer	<1	
	[Dha^{7}]MC-LR	-	-	Asp	-	-	-	-	<1	
	[X, D-Asp^{3}]MC-HilR	-	Hil	-	-	-	-	-	<1	
	[D-Asp^{3}]MC-XR	-	X	Asp	-	-	-	-	<1	
	MC-XR	-	X	-	-	-	-	-	<1	

	MC-XR	-	X	-	-	-	-	-	<1	
	MC-XR	-	X	-	-	-	-	-	<1	
	MC-XR	-	X	-	-	-	-	-	<1	
	MC-XR	-	X	-	-	-	-	-	<1	
	MC-XR	-	X	-	-	-	-	-	<1	
	MC-XR	-	X	-	-	-	-	-	<1	
	MC-XR	-	X	-	-	-	-	-	<1	
	MC-XR	-	X	-	-	-	-	-	<1	
	MC-XR	-	X	-	-	-	-	-	<1	
	MC-XR	-	X	-	-	-	-	-	<1	
	MC-XR	-	X	-	-	-	-	-	<1	
	MC-XR	-	X	-	-	-	-	-	<1	
	MC-XR	-	X	-	-	-	-	-	<1	
	MC-XR	-	X	-	-	-	-	-	<1	
	MC-XR	-	X	-	-	-	-	-	<1	
	MC-XR	-	X	-	-	-	-	-	<1	
	MC-XR	-	X	-	-	-	-	-	<1	
	MC-XR	-	X	-	-	-	-	-	<1	
	MC-XR	-	X	-	-	-	-	-	<1	
	MC-XR	-	X	-	-	-	-	-	<1	
	MC-XR	-	X	-	-	-	-	-	<1	
	MC-XR	-	X	-	-	-	-	-	<1	
Nostoc sp. 152	[ADMAdda^5]MC-LR	-	-	-	-	ADMAdda	-	-	44	1,12
	[ADMAdda^5]MC-LHar	-	-	-	Har	ADMAdda	-	-	40	
	[ADMAdda^5]MC-HilR	-	Hil	-	-	ADMAdda	-	-	1.7	
	[ADMAdda^5]MC-HilHar	-	Hil	-	Har	ADMAdda	-	-	1	
	[ADMAdda^5, Dha^7]MC-LR	-	-	-	-	ADMAdda	-	Dha	<1	
	[Ser^1, D-Asp^3,ADMAdda^5]MC-LR	Ser	-	Asp	-	ADMAdda	-	-	<1	
	[D-Asp^3,ADMAdda^5, Dha^7]MC-LR	-	-	Asp	-	ADMAdda	-	Dha	<1	
	[DMAdda^5]MC-LR	-	-	-	-	DMAdda	-	-	<1	
	[DMAdda^5]MC-LHar	-	-	-	Har	DMAdda	-	-	<1	
	[D-Asp3, DMAdda^5]MC-LR	-	-	Asp	-	DMAdda	-	-	<1	
	[ADMAdda^5]MC-XR	-	X	-	-	ADMAdda	-	-	<1	
	[D-Asp^3, ADMAdda^5]MC-VR	-	Val	Asp	-	ADMAdda	-	-	<1	
	[D-Asp^3, ADMAdda^5, Dha^7]MC-HilR	-	Hil	Asp	-	ADMAdda	-	Dha	<1	

Nostoc sp. IO-102-I	[ADMAdda^{5}]MC-LR	-	-	-	-	ADMAdda	-	-	82	1, 10
	[ADMAdda^{5}]MC-XR	-	X	-	-	ADMAdda	-	-	4	
	[D-Asp^{3}, ADMAdda^{5}]MC-LR	-	-	Asp	-	-	-	-	3	
	[DMAdda^{5}]MC-LR	-	-	-	-	DMAdda	-	-	2	
	MC-XR	-	X	-	-	-	-	-	1	
	MC-XR	-	X	-	-	-	-	-	1	
	[(X), ADMAdda^{5}]MC-(F)R	-	Phe	-	-	ADMAdda	-	-	1	
	MC-XR	-	X	-	-	-	-	-	1	
	[(X), DMAdda^{5}]MC-(Hil)R	-	Hil	-	-	DMAdda	-	-	1	
	[DMAdda^{5}]MC-HilR	-	Hil	-	-	DMAdda	-	-	<1	
	[ADMAdda^{5}]MC-XR	-	X	-	-	ADMAdda	-	-	<1	
	[ADMAdda^{5}]MC-XR	-	X	-	-	ADMAdda	-	-	<1	
	[ADMAdda^{5}]MC-YR	-	Tyr	-	-	ADMAdda	-	-	<1	
	[ADMAdda^{5}]MC-XR	-	X	-	-	ADMAdda	-	-	<1	
	[X]MC-LR	-	-	-	-	-	-	-	<1	
	MC-XR	-	X	-	-	-	-	-	<1	
	[X]MC-LR	-	-	-	-	-	-	-	<1	
	MC-XR	-	X	-	-	-	-	-	<1	
Nostoc sp. UK89IIa	[Leu^{1}]MC-LR	Leu	-	-	-	-	-	-	96	6
	[Leu^{1},Asp^{3}]MC-LR	Leu	-	Asp	-	-	-	-	2	
	[Leu^{1}]MC-HphR	Leu	Hph	-	-	-	-	-	2	
	[Leu^{1}, Dha^{7}]MC-LR	Leu	-	-	-	-	-	Dha	1	
Phormidium sp. CENA270	[Leu^{1}]MC-LR	Leu	-	-	-	-	-	-	31	6
	[Leu^{1}]MC-RR	Leu	Arg	-	-	-	-	-	31	
	[Leu^{1},Asp^{3}]MC-LR	Leu	-	Asp	-	-	-	-	18	
	[Leu^{1},Asp^{3}]MC-RR	Leu	Arg	Asp	-	-	-	-	13	
	[Leu^{1}]MC-LHar	Leu	-	-	Har	-	-	-	7	
Phormidium sp. DVL1003c	MC-LR	-	-	-	-	-	-	-	49	6,7
	MC-HphR	-	Hph	-	-	-	-	-	14	
	MC-LHty	-	-	-	-	-	-	-	12	
	MC-HphHty	-	Hph	-	Hty	-	-	-	7	
	MC-FR	-	Phe	-	-	-	-	-	7	
	MC-HtyR	-	Hty	-	-	-	-	-	4	
	[MeSer^{7}]MC-LR	-	-	-	-	-	-	MeSer	2	
	[D-Asp^{3}]MC-LHty	-	-	Asp	Hty	-	-	-	2	

	MC-LHph	-	-	-	Hph	-	-	-	1	
	MC-HphHph	-	Hph	-	Hph	-	-	-	<1	
	MC-LW	-	-	-	Trp	-	-	-	<1	
	[D-Asp^3]MC-LR	-	-	Asp	-	-	-	-	<1	
	[Dha^7]MC-LR	-	-	-	-	-	-	Dha	<1	
	[DMAdda^5]MC-LR	-	-	-	-	DMAdda	-	-	<1	
	MC-YR	-	Tyr	-	-	-	-	-	<1	
Planktothrix agardhii NIVA-CYA126/8	[D-Asp^3]MC-RR	-	Arg	Asp	-	-	-	-	92	8
	[D-Asp^3]MC-LR	-	-	Asp	-	-	-	-	7	
Planktothrix agardhii 213	[D-Asp^3]MC-RR	-	Arg	Asp	-	-	-	-	84	1
	[D-Asp^3]MC-LR	-	-	Asp	-	-	-	-	15	
	[Asp^3, Dha^7]MC-LR	-	-	Asp	-	-	-	Dha	<1	
Planktothrix rubescens NIVA-CYA98	[Dha^7]MC-LR	-	-	-	-	-	-	Dha	Ni	9
	[Dha^7]MC-RR	-	Arg	-	-	-	-	Dha	Ni	

X. MC contains an unknown amino acid or the overall amino acid content is not known. * % MC. Amounts of microcystin detected. Ni. No information.

± References:

1. Fewer DP, Rouhiainen L, Jokela J, Wahlsten M, Laakso K, Wang H, Sivonen K. 2007. Recurrent adenylation domain replacement in the microcystin synthetase gene cluster. *BMC Evol Biol.* 7:183.
2. Shirai M, Ohtake A, Sano T, Matsumoto S, Sakamoto T, Sato A, Aida T, Harada K-I, Shimada T, Suzuki M, Nakano M. 1991. Toxicity and Toxins of Natural Blooms and Isolated Strains of Microcystis spp. (Cyanobacteria) and Improved Procedure for Purification of Cultures. Appl Environ Microbiol. 57(4): 1241–1245.
3. Harada K-I, Ogawa K, Matsuura K, Nagai H, Murata H, Suzuki M, Itezono Y, Nakayama N, Shirai M, Nakano M. 1991. Isolation of two toxic heptapeptide microcystins from an axenic strain of *Microcystis aeruginosa*, K-139. Toxicon 29: 479–489.
4. Tooming-Klunderud A, Fewer DP, Rohrlack T, Jokela J, Rouhiainen L, Sivonen K, Kristensen T, Jakobsen KS. 2008. Evidence for positive selection acting on microcystin synthetase adenylation domains in three cyanobacterial genera. BMC Evol Biol. 8:256.
5. Srivastava A, Choi GG, Ahn CY, Oh HM, Ravi AK, Asthana RK. 2012. Dynamics of microcystin production and quantification of potentially toxigenic Microcystis sp. using real-time PCR. Water Res. 46:817-27.

6. This study
7. Izaguirre G, Jungblut AD, Neilan BA. 2007. Benthic cyanobacteria (Oscillatoriaceae) that produce microcystin-LR, isolated from four reservoirs in southern California. Water Res. 41(2):492-498.
8. Christiansen G, Molitor C, Philmus B, Kurmayer R. 2008. Nontoxic strains of cyanobacteria are the result of major gene deletion events induced by a transposable element. Mol. Biol. Evol. 25(8):1695–1704.
9. Rounge TB, Rohrlack T, Nederbragt AJ, Kristensen T, Jacobsen KS. 2009. A genome-wide analysis of nonribosomal peptide synthetase gene clusters and their peptides in a Planktothrix rubescens strain. BMC Genomics. 10:396.
10. Oksanen I, Jokela J, Fewer DP, Wahlsten M, Rikkinen J, Sivonen K. 2004. Discovery of Rare and Highly Toxic Microcystins from Lichen-Associated Cyanobacterium *Nostoc* sp. Strain IO-102-I. Appl Environ Microbiol. 70(10): 5756–5763.
11. Yoshida T, Yuki Y, Lei S, Chinen H, Yoshida M, Kondo R, Hiroishi S. 2003. Quantitative detection of toxic strains of the cyanobacterial genus Microcystis by competitive PCR. Microbes Environ. 18(1):16-23.
12. Sivonen, K, Namikoshi M, Evans WR, Fardig M, Carmichael WW, Rinehart KL. 1992. Three new microcystins, cyclic heptapeptide hepatotoxins, from *Nostoc* sp. strain 152. Chem. Res. Toxicol. 5:464–469.

Table S4. Primers used in this study.

Gene	Primer	Primer sequence (5’-3’)	Tm (°C)	use	Ref
mcyA	UF	TGTATTTTCCTGGGAGATGTGC	64.9	External	1
mcyA	UR	TAAATGCTATGAAAGAGCATCCC	63.0	External	1
mcyA	UF1	CTCGGCGGTTAATTCAGGTTG	60.4	Internal	1
mcyA	UF2	CTCGGCGGTTAATTCAGGTTG	61.3	Internal	1
mcyA	UF3	CTCGGCGGTTAATTCAGGTTG	60.3	Internal	1
mcyA	UF4	CTCGGCGGTTAATTCAGGTTG	55.7	Internal	1
mcyA	UR1	CTCGGCGGTTAATTCAGGTTG	58.8	Internal	1
mcyA	UR2	GTGGTGCATTTCAGCGCGA	70.9	Internal	1
mcyA	UR3	CTCGGCGGTTAATTCAGGTTG	67.4	Internal	1
mcyA	270TF1	CTCATTGCAGGATACTATC	51.8	Internal	1
mcyA	270TF2	TGTTGGATGCTATTCCACTGAC	63.8	Internal	1
mcyA	270TF3	TAACAGCTGGAGCAAGTAT	55.0	Internal	1
mcyA	270TF4	TAGAGCAGAAATTACCAACT	53.2	Internal	1
mcyA	TF5b	GGCTATTAATCAGAGTAGTGC	55.9	Internal	1
mcyA	270TR1	CGTTCTACTTGCTCTTCAAA	57.3	Internal	1
mcyA	270TR2	CTGATGACAGTAATTCCAGTC	63.0	Internal	1
mcyA	270TR3	GGTAGTTTAGTAACTGTCAGG	52.1	Internal	1
mcyA	270TR4	GATTCAAGTAATCGCTAAAGCC	60.6	Internal	1
mcyA	DVLTF1	TAGCACTGAAGAACTAGCAG	55.0	Internal	1
mcyA	DVLTF2	GTCGAGAATGCCTTAGAGTTACC	62.1	Internal	1
mcyA	DVLTR1b	CATTCATCAAGGGTTGCCTTCC	68.5	Internal	1
mcyA	DVLTR2	CAGAGCAGTTAGTAGCACGTCA	62.2	Internal	1
mcyD	mcyDF	GATCCGATTGAATTAGAAAG	55.9	External	2
mcyD	mcyDR	GTATTCCCCAAGATTGCC	59.8	External	2
mcyD	mcyDintF	GGACAAGGCTCTCAATATT	55.6	Internal	1
mcyD	mcyDintR	ATATGAGAACTCCAATCAAA	53.5	Internal	1
mcyE	mcyEF2	GAAATTTGTGTAGAAGGTGC	56.8	External	2
mcyE	mcyER4	AATTCTAAAGCCCAAAGACG	60.2	External	2

mcyE	mcyEinF	TTTAGAACSGGRGATTTAGG	53.4	Internal	1
mcyE	mcyEinR	ATTGYNGRHATGGGTTCTTGATA	58.8	Internal	1
16S rRNA	27F	AGAGTTTGATCCTGGCTCAG	51.8	External	3
16S rRNA	23S30R	CTTCGCCTCTGTGTGCCTAGGT	58.6	External	4
16S rRNA	16S545F	ATTCCGGATAACGCTTGC	63.0	Internal	5
16S rRNA	16S 979F	CGATGCAACGCGAAGAAC	66.0	Internal	5
16S rRNA	16S 1092R	GCGCTCGTTGCGGGACTT	71.3	Internal	5
16S rRNA	1494Rc	TACGGCTACCTTGTTACGAC	59.3	Internal	6

References

1. This study.
2. Rantala A, Fewer DP, Hisbergues M, Rouhiainen L, Vaitomaa J, Börner T, Sivonen K. 2004. Phylogenetic evidence for the early evolution of microcystin synthesis. *Proc. Natl. Acad. Sci. USA*. 101: 568-573.
3. Wilmotte A, Van der Auwera G, De Wachter R. 1993. Structure of the 16S ribosomal RNA of the thermophilic cyanobacterium *Chlorogloeopsis* HTF ('*Mastigocladus laminosus* HTF') strain PCC7518, and phylogenic analysis. *FEMS Lett.* 317:96-100.
4. Lepère C, Wilmotte A, Meyer B. 2000. Molecular diversity of *Microcystis* strains (Cyanophyceae, Chroococcales) based on 16S rDNA sequences. *Syst. Geogr*. 70: 275–283.
5. Rajaniemi P, Hrouzek P, Kastovska K, Willame R, Rantala A, Hoffmann L, Komarek J, Sivonen K. 2005. Phylogenetic and morphological evaluation of the genera *Anabaena*, *Aphanizomenon*, *Trichormus* and *Nostoc* (Nostocales, Cyanobacteria). *Int. J. Syst. Evol. Microbiol*. 55: 11-26.
6. Neilan BA, Jacobs D, Del Dot T, Blackall LL, Hawkins PR, Cox PT, Goodman AE. 1997. rRNA Sequences and Evolutionary Relationships among Toxic and Nontoxic Cyanobacteria of the Genus Microcystis. *Int. J. Syst. Bacteriol*. 47(3): 693-697.

Table S5. Access number of strains compared in this study. Sequences obtained in this study are in bold.

Organism	Strain	Year	Origin	*mcyD*	*mcyE*	16S	McyA	McyB	McyC	McyG	McyE
Fischerella sp.	CENA161	2004	Brazil	**JQ771634**	**JQ771640**	EU840724	-	-	-	-	-
Microcystis aeruginosa	NPLJ-4	ni	Brazil	**JQ771635**	**JQ771641**	**JQ771624**	**JQ771629**	-	-	-	-
Microcystis sp.	RST 9501	1995	Brazil	**JQ771636**	**JQ771642**	**JQ771625**	**JQ771630**	-	-	-	-
Nostoc sp.	UK89IIa	ni	Finland	**JQ771637**	**JQ771643**	**JQ771626**	**JQ771631**	-	-	-	-
Phormidium sp.	CENA270	2009	Brazil	**JQ771638**	**JQ771644**	**JQ771627**	**JQ771632**	-	-	-	-
Phormidium sp.	DVL1003c	ni	USA	**JQ771639**	**JQ771645**	**JQ771628**	**JQ771633**	-	-	-	-
Anabaena sp.	90	1986	Finland	AJ536156	AJ536156	AJ133156	AAO62586	AAO62587	AAO62588	AAO62585	AAO62582
Anabaena sp.	18B6	1986	Finland	EU151880	EU151887	EU151901	-	-	-	-	-
Anabaena sp.	66A	1986	Finland	EU151881	EU151888	AJ133157	-	-	-	-	-
Hapalosiphon hibernicus	BZ-3-1	1984	Hawaii	EU151879	EU151886	EU151900	-	-	-	-	-
Microcystis aeruginosa	UV027	ni	Israel	-	-	-	AAL82382	AAK61391	AAL82384	-	-
Microcystis aeruginosa	NIES843	1997	Japan	AP009552	AP009552	AP009552	YP_001658871	YP_001658870	YP_001658869	YP_001658877	YP_001658875
Microcystis aeruginosa	K-139	1985	Japan	-	-	-	BAA83992	BAA83993	BAA83994	BAB12213	BAB12211
Microcystis aeruginosa	PCC7806	1972	Netherlands	AM778952	AM778952	AF139299	AF183408	AAF00961	AAF00962	AAF00957	AAF00958
Microcystis aeruginosa	PCC7941	1954	Canada	AY424989	AY382536	MAU40340	-	-	-	-	-
Microcystis aeruginosa	NIES89	1981	Japan	AY424988	AY382530	MAU03403	-	-	-	-	-
Microcystis aeruginosa	B-47	ni	Japan	-	-	-	-	BAH83648	-	-	-
Microcystis aeruginosa	NIES298	ni	Japan	-	-	-	-	BAC57996	-	-	-
Microcystis viridis	NIES102	1982	Japan	EU151885	EU151892	D89033	-	-	-	-	-
Nodularia spumigena	NSOR10	1993	Australia	AY210783	AY210783	AF268014	-	-	-	-	-
Nodularia spumigena	BY1	1986	Baltic Sea	AY424987	AY817169	AF268004	-	-	-	-	-
Nodularia spumigena	HEM	1987	Baltic Sea	AY424985	AY817170	AF268005	-	-	-	-	-
Nodularia spumigena	F81	1987	Baltic Sea	AY424986	AY382542	AJ781137	-	-	-	-	-
Nostoc sp.	152	1986	Finland	EU151882	EU151889	AJ133161	**JX644441**	-	-	-	-
Nostoc sp.	IO-102-I	2000	Finland	EU151883	EU151890	AY566855	-	-	-	-	-
Nostoc sp.	CENA88	2004	Brazil	GQ259208	GQ259209	GQ259207	-	-	-	-	-
Planktothrix agardhii	213	1987	Finland	EU151884	EU151891	EU151902	-	-	-	-	-
Planktothrix agardhii	NIVA-CYA126/8	1984	Finland	AJ441056	AJ441056	AJ133166	CAD29797	CAD29798	CAD29799	CAD29795	CAD29794
Planktothrix agardhii	NIVA-CYA127	1984	Finland	AY424993	AY382553	AJ133168	-	-	-	-	-
Planktothrix rubescens	NIVA-CYA98	1982	Norway	AM990462	AM990462	AB045951	CAQ48245	CAQ48246	CAQ48247	CAQ48243	CAQ48242
Planktothrix sp.	49	1985	Finland	AY424992	AY382551	AJ133167	-	-	-	-	-

Ni = no information

Table S6. Primers designed for PCR mutagenesis.

Mutants	position	Primer	Primer sequence (5'-3')	Tm (°C)
-	-	RST PETF	CACCATGCTTTCTGAGCAGGAACAAC	64.8
-	-	RST PETR	CCCGCATACTCAGAAAGAA	54.5
G301A	301	MuG AF	AACGGTAAACCTCG**C**TGGAGAAGCTGTG	68.1
(G to A)		MuG AR	CACAGCTTCTCCA**G**CGAGGTTTACCGTT	68.1
I330T (I to T)	330	MuI TF	ACCA**C**TTGTTCCACTTTTAGCTTAGTGC	63.7
		MuI TR	GCACTAAGCTAAAAGTGGAACAA**G**TGGT	63.7
C331Y (C to Y)	331	MuC YF	ACCATTT**A**TTCCACTTTTAGCTTAGTGC	60.7
		MuC YR	GCACTAAGCTAAAAGTGGAA**T**AAATGGT	60.7
G301A, I330T, C331Y (IC to TY)*	330 and 331	MuTC YF	ACCA**C**TT**A**TTCCACTTTTAGCTTAGTGC	62.2
		MuTC YR	GCACTAAGCTAAAAGTGGAA**T**AA**G**TGGT	62.2

* The G301A mutant was used as template to construct the mutant in the three positions (G301A, I330T, C331Y).

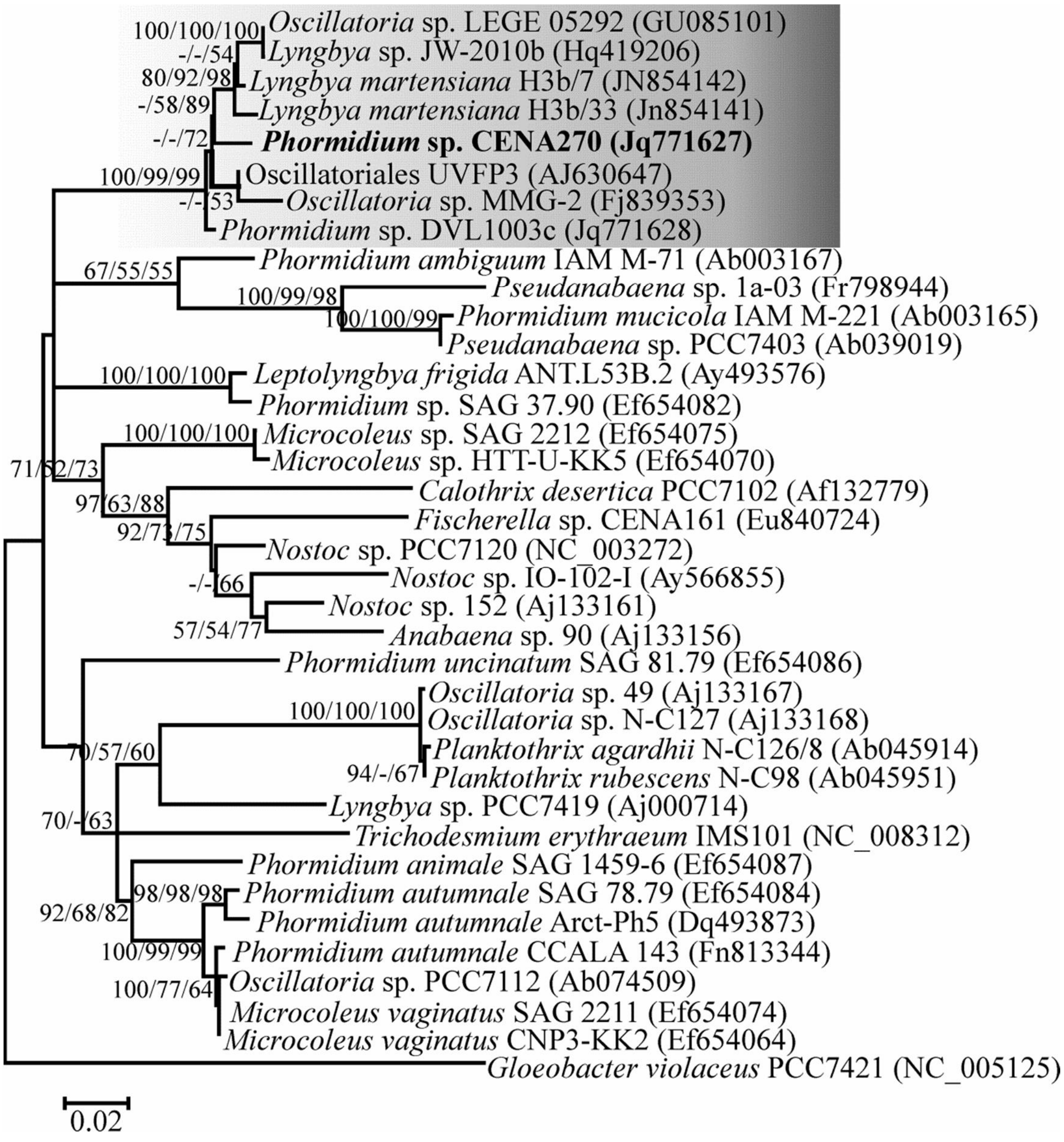

Figure S1. Phylogenetic analysis of 16S rRNA gene focusing in *Phormidium* sp. CENA270. Maximum-likelihood tree based on 16S rRNA gene. Bootstrap values above 50 per cent from 1000 respectively neighbor-joining, maximum parsimony and maximum-likelihood bootstrap replicates are given at the nodes. *Phormidium* sp. CENA270 in bold.

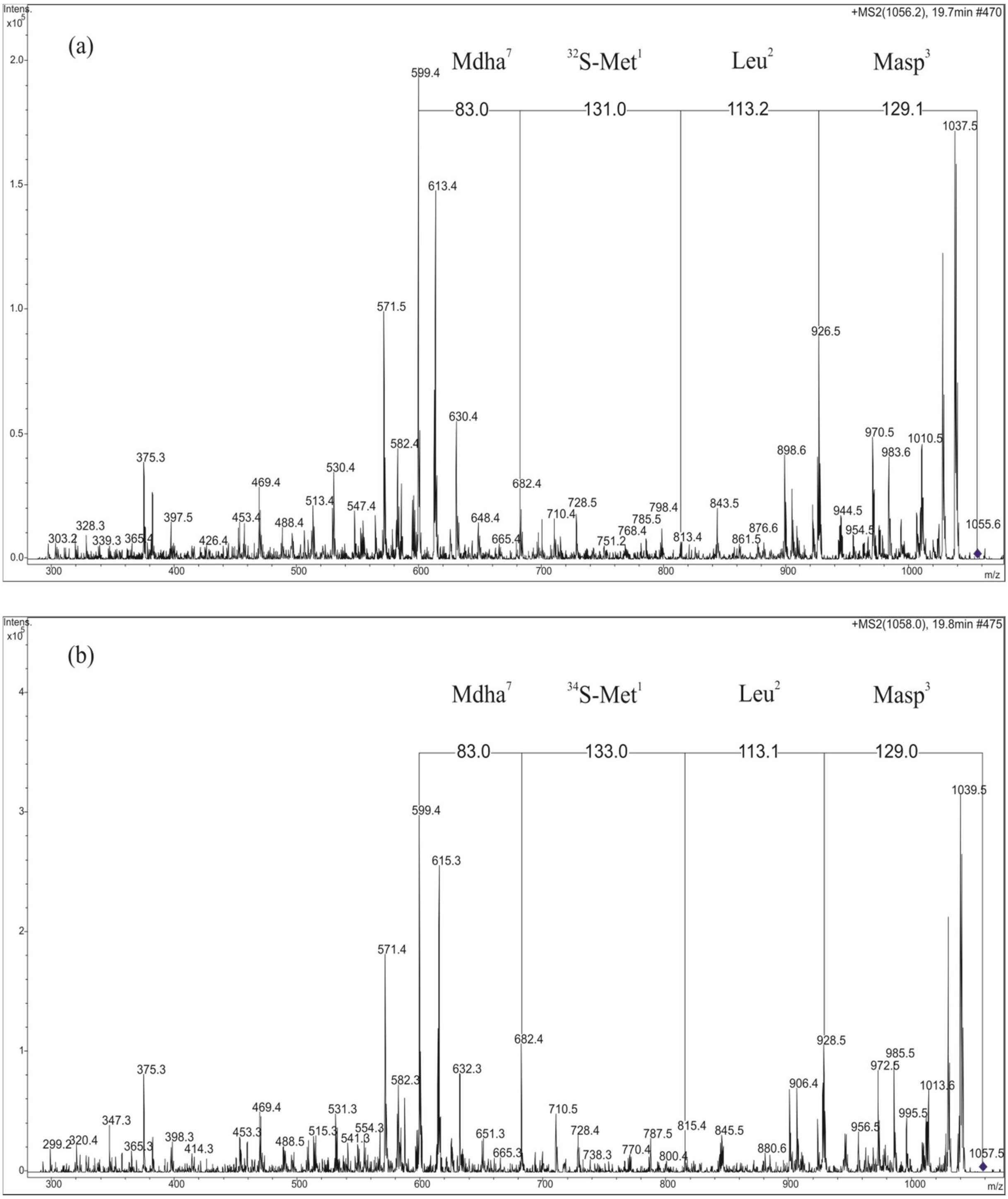

Figure S2. Product ion spectra of [Met[1]]MC-LR of *Microcystis* sp. RST 9501 in the labeling experiment. The strain was grown in two different isotope sources of the $MgSO_4.7H_2O$. (a) Control supplemented with ^{32}S. (b) Cultivation supplemented with ^{34}S.

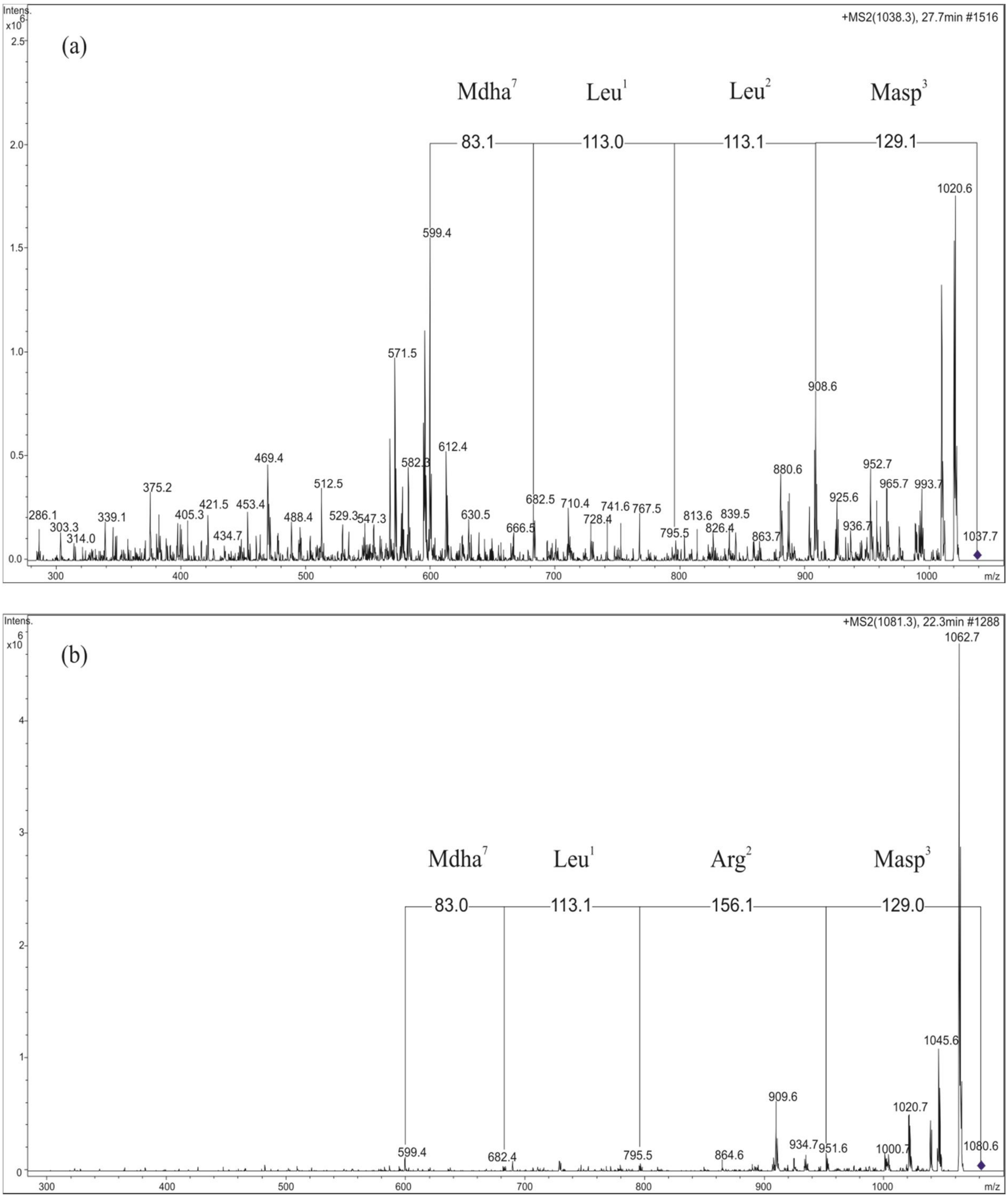

Figure S3. Product ion spectra of protonated [Leu[1]]microcystins of *Phormidium* sp. CENA270. (a) [Leu[1]]MC-LR (b) [Leu[1]]MC-RR.

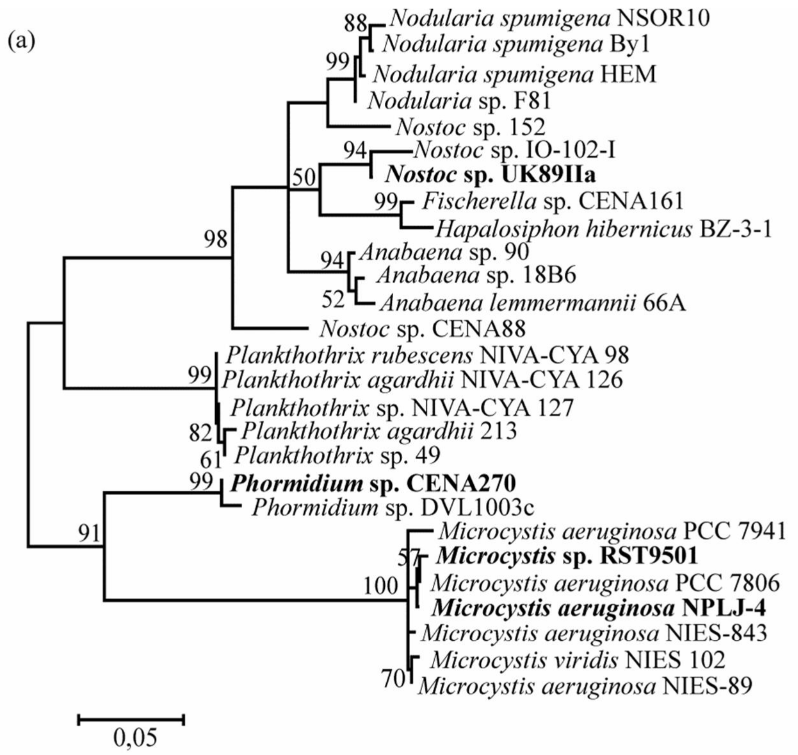

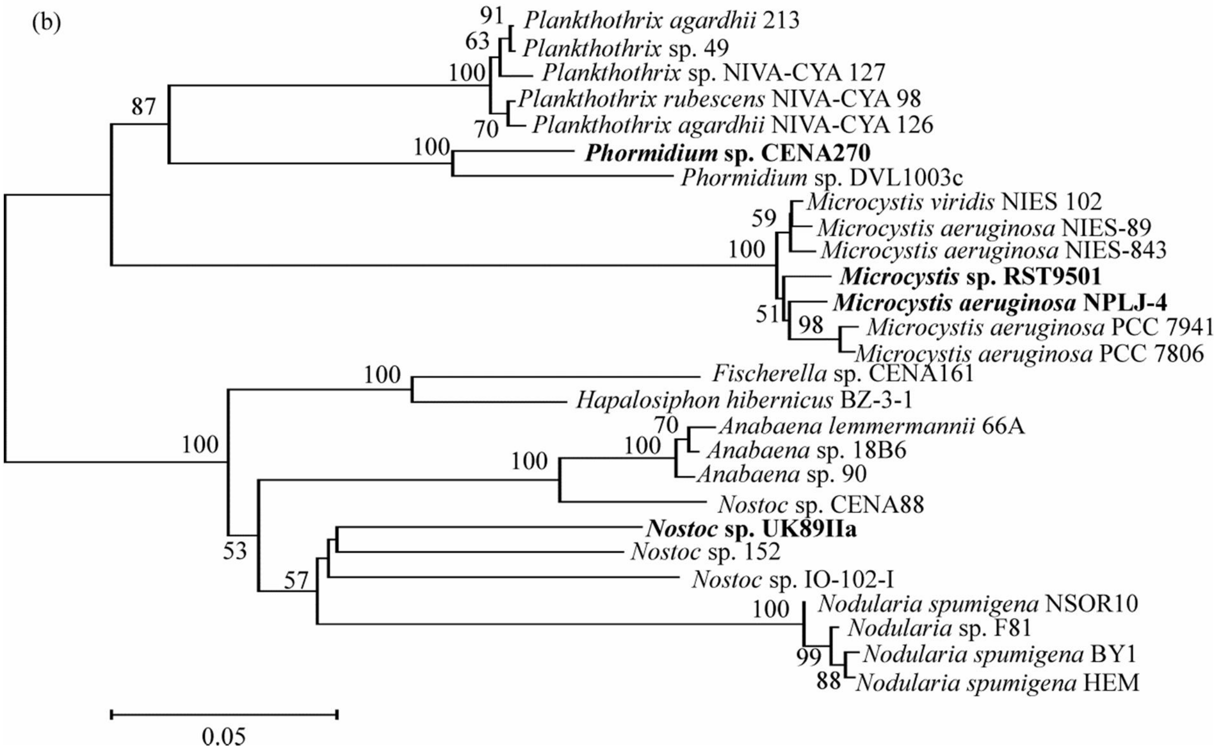

Figure S4. Phylogenetic congruence between housekeeping and microcystin synthetase genes. Maximum-likelihood tree based on (a) housekeeping (16S rRNA) and (b) microcystin synthetase (*mcyD*, *mcyE*) genes. Bootstrap values above 50 per cent from 1000 maximum-likelihood bootstrap replicates are given at the nodes. Studied strains in bold.

```
1-106
139     EQEQHQLLKEWNDTEVHYPDGCIHQLFEEQVKRSPDAIAIITENEQLTYRQLNEKANQLGHYLGKKGLKPESLVGICLERTPEMVIGLLAILKAGGAYVPLDPAYP
843     -------------------------------------------------------------------V-T------------------------------------
027     -----------------------------------------------N--P-----I---R------LNLK----------T-----------------L------
7806    ------------------------------------------------------------R--AR--V-F------------------------------------
9501    --------------------A-------------------VA------------------R--AR--V-S-----------T------------A-----------
NPLJ-4  ---------------------R-------------------A-D----------------R--AR--V-S-----------T------------------------

107-212
139     TERLNVILEDAQVSLLLTQAKLVEKLGNYPGNLVILEAEARNIALESPENLSLPVSSSNTAYVIYTSGSTGKPKGVVIEHHSTTTLLNWSKEVFSSEELAGVLGST
843     --------------------------------------------------------------------------------------------------------A-
027     -------------------E-------------------QK-----------------------------------------------------------------
7806    ---------------------------------------QK-----------------------------------------------------------------
9501    --------------V----E---------Q------A--P------------------------------------------M------G----------------
NPLJ-4  --------------V----E----------------A--P------------------------------------------M------G----------------

213-318
139     SICFDLSVFELFLPLAVGGKIILAQNVLDLPSLSAAKEVTLINTVPTAIAQLLEIEAIPETVQTVNFAGEALSNQLVQKLYQQENIKNVYNLYGPSEDTTYSTFSL
843     ------------------------------------------------------------------L---------------------------------------
027     --------------------------------------------------------------R-------------------------------------------
7806    --------------------------------------------------------------R---L---------------------------------------
9501    ---------------T---------------------------A----------------------LG---V---------------K-----------IC-----
NPLJ-4  ---------------T---------------------------A----------------------LG---V---------------K-----------IC-----

319-424
139     VPKKDHGQPSIGRPIANTQVYILDSFKQPVPLGTIGDLYIGGKGLARCYLNQPELTAEKFISNPFSNEPKAKLYKTGDLARYLPDGNIDFLGRGDNQVKLRGFRIE
843     ---GH----------------------------------------------------------------N------------------------------------
027     ---GH--------------------------------------------------------A--------------------------------------------
7806    ---GH-------------------------------------E--------------------------N------------------------------------
9501    ---GH-------------E-----------------------E--------------------------------------------------S------------
NPLJ-4  ---GH-------------------------------------E--------------------------N-----------------------S------------

425-518
139     LGEIEATLGTYPQVKQKVVKVWEDSYRNKRLVAYLVGENGPINTEDLRRFLGQKLPEYMIPALFVSLEALPLTPNGKIDRSRLPIPEIPWTSEQ
843     ---------------------------------------------------------------------------------S--L---------
027     --------------------------------------------------------------------T------------S--L---------
7806    ------------P---A-------------------A--D-------------------------------------------------S----
9501    -------I--------T--------------------------------------------------------A----------L----S----
NPLJ-4  -------I--------T--------------------------------------------------------A----------L----S----
```

Figure S5. Comparison of $McyA_2$ adenylation domain sequences from *Microcystis* strains. Yellow. A domain binding pocket; Red. Conserved sequences motifs; Green. Different amino acids from the $McyA_2$ adenylation domain activating leucine. Underlined are residues 8 Å around the substrate.

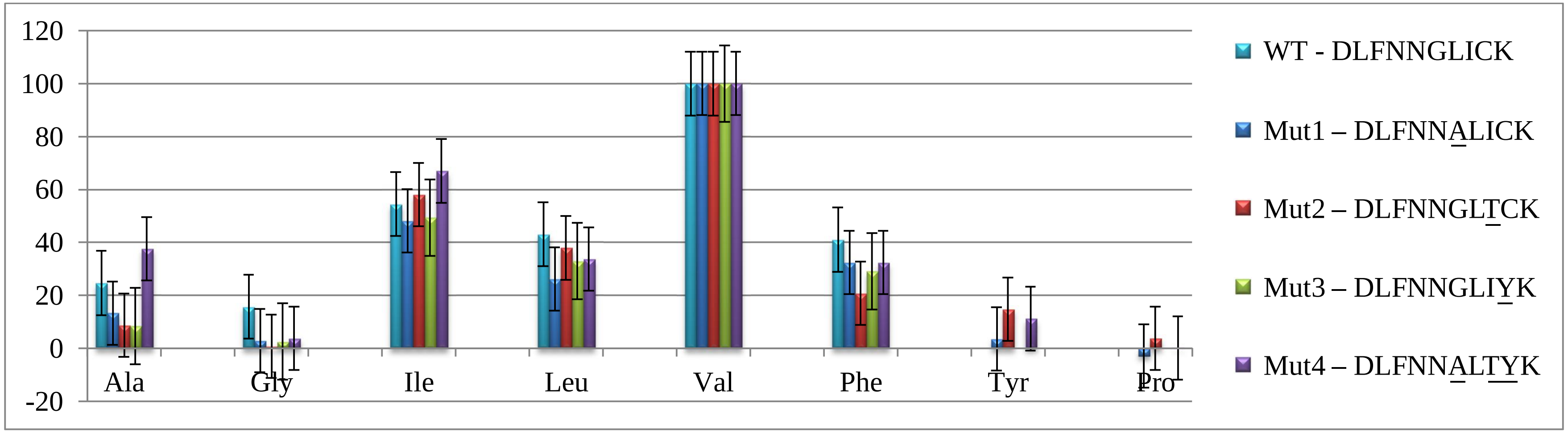

Figure S6. ATP-PPi exchange assay. This assay compared substrate specificity by adenylation domain of the McyA$_2$ from wild type (WT) in *Microcystis* sp. RST 9501 and four different mutants. Mut1. Mutant G301A contain an Ala instead of Gly in the position 301; Mut2. Mutant I330T contain a Thr instead of Ile in position 330; Mut3. Mutant C331Y contain a Tyr instead of Cys in position 331; Mut4. Mutant G301A, I330T, C331Y contain all three mutations.

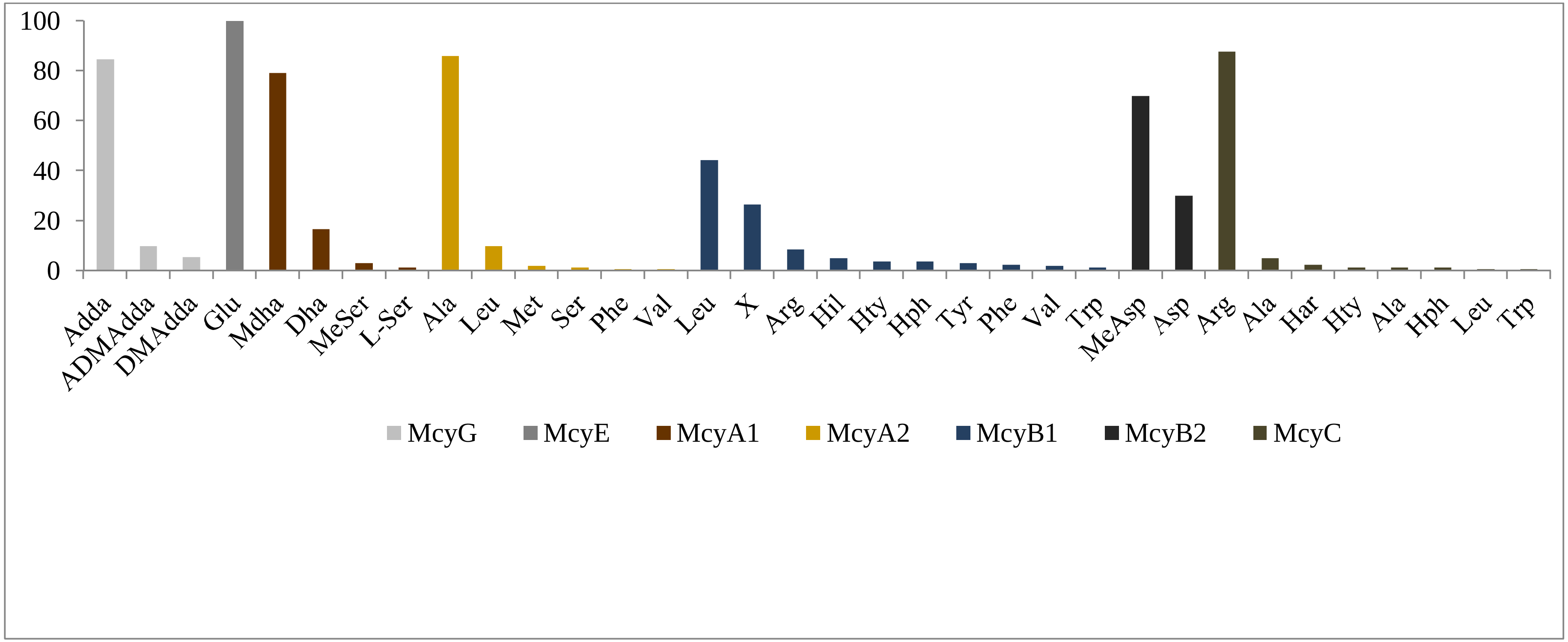

Figure S7. Relative quantity of amino acids in detected microcystins for strains included in the phylogenetic tree.
