# Supplementary material for: Convergent evolution of [D-Leucine1] microcystin-LR in taxonomically disparate cyanobacteria
Source: BMC Evol Biol. 2013 Apr 19;13:86. doi: 10.1186/1471-2148-13-86 (PMC3640908; doi:10.1186/1471-2148-13-86)
Supplement: Additional file 2: Table S1 — Assignment of the main ions from microcystin variants produced by the studied strains in LC-MS/MS. [file 1471-2148-13-86-S2.pdf]

Table S1. Assignment of main ions from microcystin variants produced by studied strains in LC-MS/MS. Ion relative intensity in superscript.

1. [Leu^{1}]MC-LR
2. [Leu^{1}, Asp^{3}] MC-LR
3. [Met^{1}]MC-LR
4. [Met^{1}, Asp^{3}]MC-LR
5. [Leu^{1}, Dha^{7}] MC-LR
6. [Leu^{1}]MC-HilR
7. [Leu^{1}]MC-HpheR
8. [Leu^{1}]MC-LHar
9. [Leu^{1}]MC-RR
10. [Leu^{1}, Asp^{3}]MC-RR

**Fragment**	***Microcystis aeruginosa* NPLJ-4**					***Microcystis* sp. RST9501**				***Nostoc* sp. UK89IIa**				***Phormidium* sp. CENA270**				
	**1.**	**2.**	**3.**	**4.**	**5.**	**1.**	**2.**	**3.**	**6.**	**1.**	**2.**	**5.**	**7.**	**1.**	**2.**	**8.**	**9.**	**10.**
M + H	**1037**	**1023**	**1055**	**1041**	**1023**	**1037**	**1023**	**1055**	**1051**	**1037**	**1023**	**1023**	**1085**	**1037**	**1023**	**1051**	**1080**	**1066**
M + H – H_2O	1019^{76}	1005^{50}	1037^{85}	1023^{22}	1005^{50}	1019^{79}	1005^{55}	1037^{100}	1033^{55}	1019^{79}	1005^{72}	1005^{72}	1067^{63}	1019^{80}	1005^{61}	1033^{64}	1062^{100}	1048^{100}
M + H – $2NH_3$	1003^{4}	989^{2}	1021^{3}	1007^{7}	989^{3}	1003^{3}	–	1021^{2}	1017^{2}	–	989^{3}	989^{6}	1051^{3}	1003^{1}	989^{2}	–	1046^{15}	1032^{9}
M + H – COOH	992^{19}	978^{12}	1010^{18}	996^{8}	978^{12}	992^{22}	978^{22}	1010^{18}	1006^{15}	992^{15}	978^{16}	978^{10}	1040^{14}	992^{22}	978^{24}	1006^{12}	–	1021^{3}
M – $(NH_2)_2C{=}NH$	978^{2}	–	996^{4}	982^{6}	964^{2}	978^{2}	964^{5}	996^{2}	992^{2}	978^{4}	–	964^{1}	1026^{3}	978^{3}	964^{2}	–	1021^{11}	1007^{7}
**b**																		
Leu*-X-(Me)Asp-(H)Arg-Adda-Glu	954^{5}	–	972^{13}	958^{15}	954^{2}	954^{6}	940^{3}	972^{7}	968^{5}	954^{8}	940^{4}	–	1002^{5}	954^{5}	940^{1}	968^{1}	–	–
Leu*-X-(Me)Asp-(H)Arg-Adda	825^{12}	811^{9}	843^{15}	829^{3}	–	825^{7}	811^{13}	843^{10}	839^{8}	825^{10}	811^{4}	–	873^{4}	825^{7}	811^{7}	839^{5}	–	–
Leu*-X-(Me)Asp-(H)Arg	512^{16}	498^{8}	530^{21}	516^{5}	–	512^{17}	498^{11}	530^{17}	526^{12}	512^{11}	498^{14}	512^{4}	560^{19}	512^{13}	498^{13}	526^{7}	–	–
X-(Me)Asp-(H)Arg-Adda-Glu-(M)dha	–	910^{5}	924^{4}	910^{6}	910^{5}	924^{7}	910^{7}	924^{2}	–	924^{7}	910^{12}	910^{7}	972^{3}	924^{5}	910^{11}	938^{3}	–	953^{1}
X-(Me)Asp-(H)Arg	399^{4}	385^{7}	399^{4}	385^{1}	399^{2}	399^{4}	385^{6}	399^{6}	413^{5}	399^{3}	385^{4}	399^{2}	447^{4}	399^{3}	385^{5}	413^{1}	–	–
(Me)Asp-(H)Arg-Adda-Glu-(M)dha-Leu*	–	910^{5}	942^{5}	928^{3}	910^{5}	924^{7}	910^{7}	942^{6}	924^{9}	924^{7}	910^{12}	910^{7}	924^{4}	924^{5}	910^{11}	938^{3}	924^{3}	910^{5}
(Me)Asp-(H)Arg-Adda-Glu	728^{14}	714^{16}	728^{10}	714^{3}	–	728^{12}	714^{9}	728^{15}	728^{17}	728^{14}	714^{9}	728^{9}	728^{17}	728^{14}	714^{10}	742^{6}	728^{3}	714^{2}
(H)Arg-Adda-Glu-(M)dha-Leu*-X	908^{41}	908^{11}	926^{44}	926^{8}	894^{23}	908^{42}	908^{11}	926^{38}	922^{26}	908^{55}	908^{11}	894^{38}	956^{50}	908^{39}	908^{17}	922^{24}	951^{5}	951^{2}
(H)Arg-Adda-Glu-(M)dha-Leu*	795^{6}	795^{9}	813^{7}	813^{3}	–	795^{8}	795^{6}	813^{10}	795^{5}	795^{7}	795^{6}	781^{3}	795^{3}	795^{5}	795^{7}	809^{4}	795^{3}	795^{1}
(H)Arg-Adda-Glu-(M)dha	682^{9}	682^{4}	682^{14}	682^{7}	668^{8}	682^{16}	682^{7}	682^{12}	682^{10}	682^{15}	682^{7}	668^{8}	682^{18}	682^{16}	682^{3}	696^{10}	682^{1}	–
(H)Arg-Adda-Glu	599^{100}	599^{100}	599^{94}	599^{100}	599^{100}	599^{100}	599^{83}	599^{99}	599^{81}	599^{100}	599^{89}	599^{100}	599^{73}	599^{80}	599^{100}	613^{100}	599^{3}	599^{2}
(H)Arg-Adda	470^{18}	470^{19}	470^{15}	470^{11}	470^{19}	470^{17}	470^{13}	470^{15}	470^{10}	470^{7}	470^{15}	470^{9}	470^{15}	470^{12}	470^{19}	484^{12}	470^{1}	–
Adda-Glu-(M)dha-Leu*-X-(Me)Asp	881^{12}	867^{2}	899^{14}	–	867^{2}	881^{15}	–	899^{14}	895^{9}	881^{21}	–	867^{7}	929^{10}	881^{11}	867^{2}	–	924^{3}	910^{5}
Glu-(M)dha-Leu*-X-(Me)Asp-(H)Arg	–	710^{2}	742^{3}	728^{4}	710^{2}	724^{2}	710^{4}	742^{2}	738^{2}	724^{6}	–	710^{10}	772^{3}	724^{2}	710^{2}	738^{1}	767^{1}	–
Glu-(M)dha-Leu*-X-(Me)Asp	568^{6}	554^{12}	586^{9}	572^{14}	554^{12}	568^{6}	554^{11}	586^{4}	582^{19}	568^{2}	554^{9}	554^{9}	616^{6}	568^{5}	554^{11}	568^{3}	–	–
(M)dha-Leu*-X-(Me)Asp-(H)Arg-Adda	908^{41}	894^{23}	926^{44}	912^{7}	894^{23}	908^{42}	894^{19}	926^{38}	922^{26}	908^{55}	894^{18}	894^{38}	956^{50}	908^{39}	894^{22}	922^{24}	951^{5}	–

(M)dha-Leu*-X-(Me)Asp-(H)Arg	595^{63}	581^{56}	613^{72}	599^{100}	581^{56}	595^{85}	581^{48}	613^{80}	609^{59}	595^{77}	581^{49}	581^{49}	643^{70}	595^{65}	581^{62}	609^{26}	–	–
(M)dha-Leu*-X-(Me)Asp	439^{6}	425^{5}	457^{12}	443^{4}	425^{5}	439^{6}	425^{10}	457^{7}	453^{10}	439^{5}	425^{7}	–	487^{5}	439^{6}	425^{6}	439^{5}	482^{1}	468^{1}
(M)dha-Leu*-X	310^{6}	310^{2}	328^{2}	328^{2}	–	310^{6}	310^{7}	328^{3}	324^{2}	310^{3}	310^{3}	296^{3}	358^{3}	310^{4}	310^{4}	310^{1}	–	–
X-(Me)Asp-(H)Arg-A_{179}	578^{5}	564^{6}	578^{5}	564^{3}	–	578^{10}	564^{10}	578^{4}	592^{5}	578^{4}	564^{9}	578^{2}	626^{9}	578^{7}	564^{4}	592^{2}	–	–
(Me)Asp-(H)Arg-A_{179}-Glu	594^{32}	580^{27}	594^{8}	580^{2}	–	594^{41}	580^{32}	594^{13}	–	594^{37}	580^{38}	–	594^{7}	594^{33}	580^{31}	608^{21}	–	–
(Me)Asp-(H)Arg-A_{179}	465^{3}	–	465^{2}	–	465^{2}	465^{7}	–	465^{3}	465^{2}	465^{5}	451^{4}	465^{6}	465^{4}	465^{4}	–	479^{3}	–	–
(H)Arg-A_{179}-Glu	465^{3}	465^{2}	465^{2}	465^{2}	465^{2}	465^{7}	465^{8}	465^{3}	465^{2}	465^{5}	465^{7}	465^{6}	465^{4}	465^{4}	465^{4}	479^{3}	–	–
A_{179}-Glu-(M)dha-Leu*-X-(Me)Asp-(H)Arg	903^{9}	889^{13}	921^{12}	907^{7}	889^{13}	903^{12}	889^{14}	921^{13}	917^{8}	903^{18}	889^{6}	889^{12}	951^{8}	903^{11}	889^{14}	917^{8}	–	–
**b - H_2O**																		
Leu*-X-(Me)Asp-(H)Arg-Adda-Glu-(M)dha	1019^{76}	1005^{50}	1037^{85}	1023^{22}	1005^{50}	1019^{79}	1005^{55}	1037^{100}	1033^{55}	1019^{79}	1005^{72}	1005^{72}	1067^{63}	1019^{80}	1005^{61}	1033^{64}	1062^{100}	1048^{100}
Leu*-X-(Me)Asp-(H)Arg-Adda-Glu	936^{6}	922^{2}	954^{4}	940^{2}	–	936^{3}	922^{7}	954^{4}	950^{4}	936^{5}	–	936^{3}	984^{4}	936^{3}	922^{3}	950^{4}	–	–
Leu*-X-(Me)Asp-(H)Arg	807^{2}	793^{3}	–	–	–	494^{5}	480^{4}	512^{4}	508^{4}	494^{11}	480^{7}	–	542^{3}	494^{4}	480^{4}	508^{4}	–	–
X-(Me)Asp-(H)Arg	381^{4}	367^{2}	–	367^{1}	–	381^{3}	367^{5}	381^{3}	395^{3}	381^{7}	367^{3}	381^{3}	429^{4}	381^{3}	367^{3}	395^{3}	–	–
(Me)Asp-(H)Arg-Adda-Glu	710^{13}	696^{10}	710^{9}	696^{2}	710^{2}	710^{16}	696^{14}	710^{12}	710^{10}	710^{12}	696^{19}	710^{10}	710^{3}	710^{14}	696^{9}	724^{7}	710^{1}	–
(Me)Asp-(H)Arg-Adda	581^{6}	567^{9}	581^{5}	567^{5}	581^{56}	581^{6}	567^{7}	581^{7}	581^{11}	581^{9}	567^{5}	581^{49}	–	581^{5}	567^{5}	595^{7}	–	–
(H)Arg-Adda-Glu-(M)dha-Leu*-X	890^{8}	890^{6}	908^{4}	908^{18}	876^{1}	890^{6}	890^{10}	908^{5}	904^{5}	890^{8}	890^{7}	876^{4}	938^{9}	890^{5}	890^{8}	904^{2}	933^{1}	–
(H)Arg-Adda-Glu	581^{6}	581^{56}	581^{5}	581^{5}	581^{56}	581^{6}	581^{48}	581^{7}	581^{11}	581^{9}	581^{49}	581^{49}	–	581^{5}	581^{62}	595^{7}	–	–
(M)dha-Leu*-X-(Me)Asp-(H)Arg-Adda	890^{8}	876^{1}	908^{4}	894^{1}	876^{1}	890^{6}	876^{5}	908^{5}	904^{5}	890^{8}	876^{6}	876^{4}	938^{9}	890^{5}	876^{3}	904^{2}	933^{1}	919^{1}
(M)dha-Leu*-X-(Me)Asp-(H)Arg	577^{10}	563^{10}	595^{16}	581^{5}	563^{10}	577^{14}	563^{10}	595^{12}	591^{9}	577^{10}	563^{11}	563^{9}	625^{29}	577^{9}	563^{9}	591^{5}	620^{1}	606^{1}
**b - NH_3**																		
Leu*-X-(Me)Asp-(H)Arg-Adda-Glu-(M)dha	1020^{94}	1006^{69}	1038^{100}	1024^{31}	1006^{69}	1020^{96}	1006^{100}	1038^{87}	1034^{68}	1020^{98}	1006^{100}	1006^{89}	1068^{100}	1020^{100}	1006^{81}	1034^{41}	–	–
Leu*-X-(Me)Asp-(H)Arg	495^{12}	481^{5}	513^{8}	499^{3}	495^{1}	495^{9}	481^{6}	513^{9}	509^{9}	495^{6}	481^{6}	495^{4}	543^{7}	495^{7}	481^{11}	509^{8}	–	–
Leu*-X-(Me)Asp	–	325^{3}	357^{5}	–	339^{2}	339^{6}	325^{3}	357^{4}	353^{4}	339^{7}	325^{5}	339^{2}	387^{11}	339^{3}	325^{3}	–	–	–
X-(Me)Asp-(H)Arg-Adda-Glu-(M)dha	907^{18}	893^{17}	907^{5}	893^{1}	893^{17}	907^{26}	893^{17}	907^{2}	921^{14}	907^{34}	893^{17}	893^{20}	955^{26}	907^{24}	893^{19}	921^{7}	–	936^{1}
X-(Me)Asp-(H)Arg	382^{14}	368^{11}	382^{9}	368^{3}	382^{2}	382^{12}	368^{4}	382^{12}	396^{8}	382^{14}	368^{7}	382^{5}	430^{15}	382^{10}	368^{7}	396^{5}	–	–
(Me)Asp-(H)Arg-Adda-Glu-(M)dha-Leu*	907^{18}	893^{17}	925^{17}	911^{9}	893^{17}	907^{26}	893^{17}	925^{30}	–	907^{34}	893^{17}	893^{20}	907^{2}	907^{24}	893^{19}	921^{7}	907^{1}	893^{2}
(Me)Asp-(H)Arg-Adda-Glu	711^{4}	697^{7}	711^{5}	697^{3}	–	711^{8}	697^{6}	711^{6}	711^{8}	711^{9}	697^{13}	711^{9}	711^{5}	711^{6}	697^{6}	725^{2}	711^{1}	697^{1}
(Me)Asp-(H)Arg-Adda	582^{21}	568^{5}	582^{20}	568^{4}	582^{25}	582^{19}	568^{3}	582^{22}	582^{19}	582^{14}	–	582^{26}	582^{19}	582^{17}	568^{2}	596^{4}	582^{1}	–
(H)Arg-Adda-Glu-(M)dha-Leu*-X	891^{3}	891^{6}	909^{3}	909^{21}	877^{2}	891^{5}	891^{15}	909^{5}	905^{3}	891^{9}	891^{5}	877^{3}	939^{2}	891^{4}	891^{5}	905^{1}	934^{3}	934^{1}
(H)Arg-Adda-Glu	582^{21}	582^{25}	582^{20}	582^{14}	582^{25}	582^{19}	582^{28}	582^{22}	582^{19}	582^{14}	582^{29}	582^{26}	582^{19}	582^{17}	582^{35}	596^{4}	582^{1}	582^{1}
(H)Arg-Adda	453^{7}	453^{10}	453^{8}	453^{3}	453^{10}	453^{10}	453^{6}	453^{6}	453^{10}	453^{11}	453^{17}	453^{6}	453^{4}	453^{6}	453^{7}	467^{1}	–	–
Adda-Glu-(M)dha	509^{3}	509^{4}	509^{3}	509^{3}	495^{1}	509^{5}	–	509^{5}	509^{9}	509^{4}	509^{2}	495^{4}	509^{3}	509^{4}	509^{6}	509^{8}	–	–
Adda-Glu	426^{4}	426^{2}	–	–	426^{2}	426^{2}	426^{2}	426^{2}	426^{1}	426^{2}	426^{7}	426^{2}	426^{2}	426^{2}	426^{6}	426^{2}	–	–
(M)dha-Leu*-X-(Me)Asp-(H)Arg	578^{5}	564^{6}	596^{7}	582^{14}	564^{6}	578^{10}	564^{10}	596^{8}	592^{5}	578^{4}	564^{9}	564^{24}	626^{9}	578^{7}	564^{4}	592^{2}	–	–
(Me)Asp-(H)Arg-A_{179}-Glu	577^{10}	563^{10}	577^{2}	–	–	577^{14}	563^{10}	577^{1}	577^{3}	577^{10}	563^{11}	–	577^{12}	577^{9}	563^{9}	591^{5}	–	–
A_{179}-Glu-(M)dha-Leu*-X-(Me)Asp-(H)Arg	886^{19}	872^{16}	904^{11}	890^{10}	872^{16}	886^{19}	872^{17}	904^{22}	900^{11}	886^{12}	872^{14}	872^{10}	934^{9}	886^{18}	872^{21}	900^{2}	–	–

A$_{179}$-Glu-(M)dha-Leu*	488^{14}	488^{10}	–	506^{2}	–	488^{15}	488^{9}	506^{4}	488^{7}	488^{9}	488^{7}	474^{7}	488^{12}	488^{10}	488^{11}	488^{5}	–	488^{1}
A$_{179}$-Glu-(M)dha	375^{20}	375^{23}	375^{17}	375^{11}	–	375^{18}	375^{22}	375^{26}	375^{19}	375^{26}	375^{10}	361^{27}	375^{13}	375^{23}	375^{27}	375^{12}	–	–
**b - H_2O - NH_3**																		
Leu*-X-(Me)Asp-(H)Arg	477^{9}	–	495^{6}	–	477^{5}	477^{5}	–	495^{5}	491^{2}	477^{8}	463^{2}	477^{4}	525^{3}	477^{4}	463^{4}	491^{2}	–	–
(Me)Asp-(H)Arg-Adda-Glu	693^{5}	679^{2}	693^{4}	679^{3}	–	693^{3}	679^{3}	693^{3}	693^{5}	–	679^{2}	693^{6}	693^{4}	693^{2}	679^{2}	707^{2}	–	–
(Me)Asp-(H)Arg-Adda	564^{8}	550^{6}	564^{7}	550^{2}	564^{6}	564^{7}	550^{8}	564^{6}	564^{2}	564^{8}	550^{5}	564^{24}	–	564^{5}	550^{6}	578^{2}	–	–
(H)Arg-Adda-Glu	564^{8}	564^{6}	564^{7}	564^{3}	564^{6}	564^{7}	564^{10}	564^{6}	564^{2}	564^{8}	564^{9}	564^{24}	–	564^{5}	564^{4}	578^{2}	–	–
**b – NH=C=NH**																		
Leu*-X-(Me)Asp-(H)Arg-Adda-Glu-(M)dha	–	–	–	999^{10}	–	–	981^{6}	1013^{3}	1009^{3}	–	–	981^{3}	–	995^{1}	981^{2}	–	1038^{9}	1024^{5}
(H)Arg-Adda-Glu-(M)dha-Leu*-X	–	866^{3}	884^{2}	884^{1}	852^{1}	–	–	–	–	–	866^{3}	852^{2}	–	–	866^{2}	–	909^{12}	909^{5}
Glu-(M)dha-Leu*-X-(Me)Asp-(H)Arg	682^{9}	668^{8}	700^{4}	–	668^{8}	682^{16}	668^{10}	700^{5}	–	682^{15}	668^{7}	668^{8}	730^{6}	682^{16}	668^{13}	–	–	–
**b + NH_3**																		
Leu*-X-(Me)Asp-(H)Arg	529^{4}	515^{3}	547^{10}	533^{4}	–	529^{9}	515^{10}	547^{8}	543^{5}	529^{6}	515^{3}	529^{16}	577^{12}	529^{5}	515^{4}	543^{2}	–	–
X-(Me)Asp-(H)Arg-Adda-Glu	858^{2}	844^{2}	858^{2}	–	–	858^{3}	844^{5}	858^{3}	872^{3}	858^{4}	844^{7}	–	906^{6}	858^{4}	844^{4}	872^{1}	–	–
(Me)Asp-(H)Arg	858^{2}	844^{2}	858^{2}	–	–	303^{5}	–	303^{6}	303^{4}	303^{7}	289^{4}	303^{4}	303^{3}	303^{4}	289^{3}	317^{2}	–	–
(H)Arg-Adda-Glu-(M)dha-Leu*-X	925^{9}	925^{8}	943^{4}	943^{4}	911^{3}	925^{10}	925^{5}	943^{8}	939^{3}	925^{6}	925^{11}	911^{2}	973^{4}	925^{9}	925^{9}	939^{9}	968^{1}	–
(M)dha-Leu*-X-(Me)Asp-(H)Arg-Adda	925^{9}	911^{3}	943^{4}	929^{2}	911^{3}	925^{10}	911^{7}	943^{8}	939^{3}	925^{6}	911^{4}	911^{2}	973^{4}	925^{9}	911^{5}	939^{9}	968^{1}	–
(M)dha-Leu*-X-(Me)Asp-(H)Arg	612^{28}	598^{22}	630^{28}	616^{10}	598^{22}	612^{28}	598^{23}	630^{26}	626^{25}	612^{24}	598^{24}	598^{28}	660^{32}	612^{26}	598^{26}	626^{12}	–	–
X-(Me)Asp-(H)Arg-A$_{179}$	595^{63}	581^{56}	595^{16}	581^{5}	–	595^{85}	581^{48}	595^{12}	609^{59}	595^{77}	581^{49}	–	643^{70}	595^{65}	581^{62}	609^{26}	–	–
**b - CO**																		
Leu*-X-(Me)Asp-(H)Arg-Adda-Glu-(M)dha	1009^{59}	995^{47}	1027^{61}	1013^{20}	995^{47}	1009^{67}	995^{63}	1027^{76}	1023^{59}	1009^{87}	995^{38}	995^{41}	1057^{50}	1009^{62}	995^{69}	1023^{44}	–	–
Leu*-X-(Me)Asp-(H)Arg-Adda-Glu	926^{9}	912^{3}	944^{10}	–	926^{13}	926^{12}	–	944^{13}	940^{6}	926^{9}	912^{3}	926^{2}	974^{14}	926^{9}	912^{1}	940^{5}	969^{1}	–
(Me)Asp-(H)Arg-Adda-Glu	700^{5}	686^{4}	700^{4}	–	–	700^{5}	–	700^{5}	700^{3}	700^{5}	686^{5}	700^{3}	700^{7}	700^{3}	686^{4}	714^{3}	–	–
(H)Arg-Adda-Glu-(M)dha-Leu*-X	880^{22}	880^{14}	898^{14}	898^{5}	866^{3}	880^{21}	880^{11}	898^{25}	894^{15}	880^{14}	880^{6}	866^{10}	928^{27}	880^{17}	880^{18}	894^{21}	923^{1}	–
(H)Arg-Adda-Glu-(M)dha-Leu*	767^{10}	767^{10}	785^{5}	–	753^{5}	767^{8}	767^{5}	785^{9}	767^{4}	767^{7}	767^{5}	753^{2}	767^{7}	767^{8}	767^{4}	781^{7}	767^{1}	767^{1}
(H)Arg-Adda-Glu	571^{45}	571^{45}	571^{49}	571^{25}	571^{45}	571^{54}	571^{45}	571^{58}	571^{35}	571^{48}	571^{21}	571^{44}	571^{53}	571^{39}	571^{50}	585^{47}	571^{1}	571^{1}
Adda-Glu-(M)dha-Leu*-X	–	–	742^{3}	742^{1}	710^{2}	724^{2}	724^{6}	742^{2}	738^{2}	724^{6}	–	710^{10}	772^{3}	724^{2}	–	724^{7}	767^{1}	767^{1}
(M)dha-Leu*-X-(Me)Asp-(H)Arg-Adda	880^{22}	866^{3}	898^{14}	884^{1}	866^{3}	880^{21}	–	898^{25}	894^{15}	880^{14}	866^{3}	866^{10}	928^{27}	880^{17}	866^{2}	894^{21}	923^{1}	909^{5}
(M)dha-Leu*-X-(Me)Asp-(H)Arg	567^{11}	553^{13}	585^{8}	571^{25}	553^{13}	567^{20}	553^{9}	585^{11}	581^{11}	567^{9}	553^{8}	553^{12}	615^{17}	567^{9}	553^{10}	581^{6}	–	–
X-(Me)Asp-(H)Arg-A$_{179}$-Glu	679^{4}	665^{8}	679^{6}	665^{2}	679^{2}	679^{7}	665^{3}	679^{3}	693^{5}	679^{5}	–	679^{1}	727^{8}	679^{6}	665^{6}	693^{2}	–	–
X-(Me)Asp-(H)Arg-A$_{179}$	550^{3}	536^{2}	550^{4}	–	550^{6}	550^{3}	–	550^{1}	564^{2}	550^{10}	536^{4}	550^{1}	598^{8}	550^{4}	536^{2}	564^{1}	–	–
A$_{179}$-Glu-(M)dha-Leu*	477^{9}	477^{5}	495^{6}	495^{3}	–	477^{5}	477^{3}	495^{5}	477^{2}	477^{8}	477^{7}	463^{2}	477^{2}	477^{4}	477^{2}	477^{1}	477^{1}	–
**b - CO - H_2O**																		
Leu*-X-(Me)Asp-(H)Arg-Adda-Glu	908^{41}	894^{23}	926^{44}	912^{7}	908^{11}	908^{42}	894^{19}	926^{38}	922^{26}	908^{55}	894^{18}	–	956^{50}	908^{39}	894^{22}	922^{24}	951^{5}	–

(Me)Asp-(H)Arg-Adda-Glu	682^{9}	668^{8}	682^{14}	668^{5}	682^{4}	682^{16}	668^{10}	682^{12}	682^{10}	682^{15}	668^{7}	682^{12}	682^{18}	682^{16}	668^{13}	696^{10}	682^{1}	–
(Me)Asp-(H)Arg-Adda	553^{5}	539^{4}	553^{8}	539^{2}	553^{13}	553^{4}	539^{9}	553^{3}	–	553^{6}	539^{5}	553^{12}	553^{6}	553^{4}	539^{6}	567^{2}	–	–
(H)Arg-Adda-Glu	553^{5}	553^{13}	553^{8}	553^{4}	553^{13}	553^{4}	553^{9}	553^{3}	–	553^{6}	553^{8}	553^{12}	553^{6}	553^{4}	553^{10}	567^{2}	–	–
Adda-Glu	397^{5}	397^{5}	397^{16}	397^{4}	397^{5}	397^{8}	397^{20}	397^{11}	397^{11}	397^{9}	397^{14}	397^{3}	397^{10}	397^{7}	397^{14}	–	397^{1}	–

Leu* = Leucine, except in microcystin [Met^{1}]MC-LR and [Met^{1}, Asp^{3}]MC-LR

X = variable amino acid in position two: Leu, Hil, Hph or Arg

A_{179} = $C_{11}H_{17}NO$ = Adda - 134

b-ion = formed from the cleavage of peptide bonds
