# Supplementary material for: Convergent evolution of [D-Leucine1] microcystin-LR in taxonomically disparate cyanobacteria
Source: BMC Evol Biol. 2013 Apr 19;13:86. doi: 10.1186/1471-2148-13-86 (PMC3640908; doi:10.1186/1471-2148-13-86)
Supplement: Additional file 3 — Supplementary information in Methods. Additional file 3 is a PDF file that contains further information to complement the Methods sub-sections: Sequencing and phylogenetic analysis; McyA2 adenylation domain mutation and heterologous expression; and Chemical analysis. [file 1471-2148-13-86-S3.pdf]

Supplementary information in Methods

Sequencing and phylogenetic analysis

The 16S rRNA, *mcyA*, *mcyD* and *mcyE* genes were amplified in PCR with final volume of 50 µL containing 1x PCR buffer supplemented with 1.5 mM $MgCl_2$, 200 µM of each dNTP, 1 µM of each external forward and reverse primers (supplementary table S1, Supplementary Material online), 2 U of Dynazyme™ II DNA polymerase (Finnzymes) and template DNA (9-100 ng μL^{-1}). The PCR thermocycle for amplifying the 16S rRNA gene consisted of initial denaturation at 94 °C for 5 min, 38 cycles of denaturation at 94 °C for 30 s, annealing at 52 °C for 30 s and extension at 72 °C for 2 min, followed by final extension at 72 °C for 15 min. The PCR thermocycle for the *mcyD* and *mcyE* genes consisted of an initial denaturation at 94 °C for 3 min, 38 cycles of denaturation at 94 °C for 30 s, annealing at 54 °C for 30 s and extension at 72 °C for 1 min, followed by final extension at 72 °C for 10 min. The *mcyA* gene was amplified in PCR with final volume of 20 µL containing 1x PCR buffer supplemented with 1.5 mM $MgCl_2$, 800 µM of each dNTP, 1 µM of each external forward and reverse primers (supplementary table S1, Supplementary Material online), 1.5 U of SUPER TAQ *plus* DNA polymerase (Enzyme Technologies Limited, HT Biotechnology LTD) and template DNA (9-100 ng μL^{-1}). The PCR thermocycle for amplifying the 16S rRNA gene consisted of initial denaturation at 94 °C for 5 min, 38 cycles of denaturation at 94 °C for 30 s, annealing at 60 °C for 30s and extension at 68 °C for 6 min, followed by final extension at 68 °C for 15 min. The fragments were verified in 0.7 % (w/v) of agarose gel.

McyA$_2$ adenylation domain mutation and heterologous expression

PCR reaction: The adenylation domain was amplified in PCR with final volume of 50 µL containing 1x PCR buffer supplemented with 2.5 mM $MgSO_4$, 200 µM of each dNTP, 1 µM of each external RSTPETF and RSTPETR, 2.5 U of *Pfu* DNA polymerase (Fermentas) and genomic DNA (9-100 ng µL^{-1}). The thermocycle consisted of an initial denaturation at 94 °C for 3 min, 25 cycles of denaturation at 94 °C for 30 s, annealing at 52 °C for 30 s and extension at 72 °C for 2 min, followed by final extension at 72 °C for 10 min.

Site-direct mutation: Mutants were obtained by site-directed mutation using PCR mutagenesis consisted of two reactions. In the first reaction, the mutation was generated using specific primers with the mutation inserted in its sequence (supplementary table S3, Supplementary Material online) and the primers RST PETF or RST PETF. The second reaction was performed to ligate the two fragments from first reaction and RST PETF and RST PETF primers were used. The reaction was performed using *Pfu* DNA polymerase (Fermentas) as described above containing 130 ng of plasmid. The second PCR was performed using primers RST PETF and RST PETR for ligation of the two products obtained in the first reaction. Mutated and wild type adenylation domains were cloned into pFN18A (HaloTag® 7) T7 Flexi® vector (Promega, WI, USA) and transformed in *Escherichia coli* KRX competent cells (Promega) competent cells following the manufacture instructions. The transformants were checked by PCR for containing inserts with the right size, and the correct positioning of the inserts was confirmed by cycle sequencing. The plasmids with the right clones were transformed in *Escherichia coli*. Transformants were grown in shaker (160 RPM) at 37 °C overnight in 3 mL of LB medium with carbenicillin (50 µg ml^{-1}) and glucose (0.05 %, w/v). In the following day, 400 µL was inoculated in 20 mL of 2xYT medium with carbenicillin (50

µg ml^{-1}) and glucose (0.05 %, w/vol) and incubated shaking for 1.5 h at 37 °C. Heterologous expression of the adenylation domains was induced by adding rhamnose to 0.1 % and the culture were grown overnight (16-18) at 24 °C with shaking (100RPM). Soluble protein expression was confirmed in 10 % SDS PAGE gel. For purification the HaloTag® Protein Purification System (Promega) was used.

Chemical analysis

Identification of microcystin variants was performed using the Agilent 1100 Series LC/MSD Trap XCT Plus high-performance liquid chromatograph mass spectrometer (Agilent Technologies). Freeze-dried biomass (150-200 mg) from each strain was extracted with 1 mL of methanol by shaking three times with 0.5 mm glass beads in FastPrep homogenizer (M.P. Biomedicals) at 6.5 m s^{-1} for 20 seconds. The extracts were subjected to centrifugation (10,000 x g, 5 min, 25 °C) and 10 µL of the extract was injected to a Luna C18(2) column (150x2.1 mm, 5µm, Phenomenex) for the LC-MS/MS analysis. The mobile phase was composed of 0.1% formic acid in water (A) and isopropyl alcohol (B). The column was eluted in a linear gradient from 5 to 65 % of B in 40 min at a flow rate of 0.15 mL min^{-1} at 40 °C. The extract of ^{34}S-labeled and unlabeled *Microcystis* sp. RST 9501 cells were eluted in a gradient from 20 to 70 % of B at a flow rate of 0.2 mL min^{-1} at 30 °C. Electrospray ionization in positive mode was used and the product ion spectra of protonated microcystins were analyzed to identify the structure of the variant.

The remains of the methanol extracts were mixed with water and dichloromethane in equivalent proportions (1:1:1). The hydrophilic upper phase was diluted with water and

passed through a preconditioned SPE-column (strata 8B-S100-UBJ, Phenomenex). Microcystin containing fractions were recovered in 1 mL of methanol. Microcystins were isolated and purified with HPLC (HP 1100 Series modular chromatograph, Agilent Technologies). Extracts were passed through a Luna C18(2) column (150x4.6 mm, 5 um, Phenomenex) with mobile phase of 0.5 % TFA in water and acetonitrile in a gradient from 20 to 50 % of acetonitrile in 45 min. The microcystins fractions were evaporated with vacuum centrifuge in glass vials and then hydrolyzed in sealed 1 mL vials with 200 µL of 6 M of deuterium chloride (catalog no. 543047; 35 wt % solution in D_2O, 99 atom % D, Sigma-Aldrich) 24h in heating oven at 110 °C. Solutions were evaporated in vacuum centrifuge and hydrolysates were prepared for enantiomeric amino acid analysis using FDAA (Pierce) as a Marfey reagent as described previously (Jokela et al. 2010). The reaction was stopped with 1 N HCl, and the solution was dried in vacuum centrifuge. The residue was dissolved to 200 µL of 50 % of acetone in water and the precipitates were centrifuged (10,000 x g, 5 min, 25 °C) away. Chiral analysis of the amino acids alanine, leucine, arginine and homoarginine was carried out with the LC-MS/MS. Amino acids from the common [*D*-Ala^1]MC-LR and –RR variants produced by *Anabaena* sp. 90 were analyzed as references. Luna C18(2) column (150x2.1 mm, 5µm, Phenomenex) was used. The mobile phase was composed of 0.1% formic acid in water (A) and acetonitrile (B). Samples were eluted in a gradient from 10 to 85 % of B in 60 min at a flow rate of 0.2 mL min^{-1} at 35 °C. Microcystin variants which had Leu in both positions 1 and 2 according to the product ion spectra contain both *L*- and *D*-Leu in molar ratio of 1:1 (range 42-48:58-52) (Figures 1a and b; Additional file 3: Table S2). When Leu was found in position 1 or 2 only *D*- or *L*-Leu was found (Additional file 3: Table S2). Both *L*- and *D*-Leu were absent when a control [Ala^1]MC-RR was analyzed (Additional file 3: Table S2). Acid hydrolysis was

performed with deuterated reagents (DCl, D_2O) which enabled recognition of amino acid enantiomers produced through racemisation reaction. *L*- or *D*- amino acids formed through this reaction were found only in trace amounts and so the detected amino acid enantiomers were original constituents of the corresponding microcystin variants.
